# Natural variation in *PtobZIP18* confers the trade‐off between stem growth and drought tolerance in *Populus*

**DOI:** 10.1111/pbi.70261

**Published:** 2025-07-13

**Authors:** Zhuoying Jin, Peng Li, Rui Huang, Lianzheng Li, Mengjiao Zhang, Donghai Zhang, Mingjia Yuan, Jintao Du, Jiaxuan Zhou, Wenke Zhang, Liang Du, Li Ji, Mingyang Quan, Deqiang Zhang, Li‐Jun Liu, Qingzhang Du

**Affiliations:** ^1^ State Key Laboratory of Tree Genetics and Breeding, College of Biological Sciences and Technology Beijing Forestry University Beijing China; ^2^ National Engineering Research Center of Tree Breeding and Ecological Restoration, College of Biological Sciences and Technology Beijing Forestry University Beijing China; ^3^ Key Laboratory of Genetics and Breeding in Forest Trees and Ornamental Plants, Ministry of Education, College of Biological Sciences and Technology Beijing Forestry University Beijing China; ^4^ College of Forestry, State Forestry and Grassland Administration Key Laboratory of Silviculture in downstream areas of the Yellow River Shandong Agricultural University Tai'an Shandong China

**Keywords:** *Populus*, natural variation, stem growth, vessel structure, water transport capacity, phosphorylation

## Abstract

Maintaining the balance between growth and drought tolerance is arguably one of the most prevalent challenges encountered by woody plants. In this study, we performed genome‐wide association studies (GWAS) of percentage loss of diameter (PLD) in the stems of 300 *Populus tomentosa* accessions under drought stress. Our analysis identified the *bZIP* transcription factor *PtobZIP18* as a key regulator of xylem development in response to drought stress. *PtobZIP18* directly increased the expression of *PtoGATL3*, *PtoCESA3* and *PtoDUF1635*, thereby influencing wood composition and vessel density. Under well‐watered conditions, *PtobZIP18* regulated the formation of significantly larger stem diameters. Conversely, PtoCIPK9 and *PtoWRKY19* synergistically reduced *PtobZIP18* protein levels by modulating its stability and transcription, thereby regulating water transport capacity under drought stress. Furthermore, a 110‐bp structural variation (SV) and three single‐nucleotide polymorphisms (SNPs) in the *PtobZIP18* promoter divided the natural population into two haplotypes (*PtobZIP18* ^*hap1*^ and *PtobZIP18* ^*hap2*^). The upstream regulator PtoWRKY19 exhibited different binding affinities to these two haplotypes, resulting in differential transcriptional responses. These variations were correlated with distinct adaptive xylem structures under drought stress across three climatic regions. We further evaluated the inheritance stabilization and breeding potential of *PtobZIP18* ^*hap1*^ and *PtobZIP18* ^*hap2*^ by using 30 hybridization populations at two latitudinal locations. Our findings imply that *PtobZIP18* ^*hap1*^ confers advantages for production‐related applications, whereas *PtobZIP18* ^*hap2*^ enhances drought resistance, providing insights into tree precision breeding aimed at optimizing growth or improving drought tolerance.

## Introduction

Global increases in tree mortality and drought‐induced forest dieback have been extensively documented across diverse ecosystems (Allen *et al*., 2010). These trends are projected to intensify as climate change amplifies the frequency and severity of drought events (IPCC, 2022). In long‐lived woody perennials, particularly large‐statured trees, the xylem's vessel role as both hydraulic conduit and structural scaffold becomes critically vulnerable under intensifying drought regimes. This vascular tissue orchestrates three essential functions—sustaining transpiration‐driven water transport, stabilizing massive trunk architectures, and buffering hydraulic fluctuations through water storage (Couvreur *et al*., 2018; Pratt *et al*., 2021; van der Sande *et al*., 2019). Drought, as a significant environmental stressor, can compromise the structural integrity of the xylem, leading to hydraulic dysfunction and a reduction in water transport capacity. This impairment compromises the plant's ability to sustain transpiration‐driven water flow, thereby limiting carbon assimilation and overall physiological performance (Anderegg *et al*., 2011; Nardini *et al*., 2010). In severe cases, prolonged drought triggers irreversible xylem collapse, culminating in tree mortality. Under drought stress, the xylem structure and function of plants must adapt to optimize water use efficiency and enhance mechanical support (Hacke *et al*., 2001; Li *et al*., 2019). These processes enable effective water transport under water‐limited conditions to be maintained in plants, while simultaneously strengthening mechanical support to ensure overall stability.

However, maintaining these essential functions, particularly ensuring hydraulic safety (to avoid lethal embolism) and mechanical stability, often comes at a significant resource cost and forms a fundamental trade‐off with the plant's growth potential. This ‘growth‐stress resistance’ trade‐off lies at the heart of plant adaptive strategies. In water‐abundant environments, plants prioritize water investment in rapid growth; however, this strategy is often accompanied by increased sensitivity to hydraulic failure and mechanical damage. Under drought stress, trees often need to prioritize the allocation of limited resources to defensive structures and functions, which inevitably diverts resources that could otherwise be used for growth, resulting in a reduced growth rate. Plants dynamically reconfigure xylem structure through adaptive strategies such as modulating vessel size‐frequency trade‐offs to balance water transport efficiency and embolism resistance (Li *et al*., 2019; Liu *et al*., 2022) larger vessels enhance hydraulic conductivity under well‐watered conditions, their increased vulnerability to drought‐induced embolism poses catastrophic failure risks (Avila *et al*., 2023; Bauerle *et al*., 2011). Conversely, smaller vessels improve embolism resistance but limit maximum water transport capacity (Li *et al*., 2019; Liu *et al*., 2022a). Furthermore, reinforcing mechanical stability via lignin deposition and secondary cell wall remodelling (Arend and Fromm, 2007; Yu *et al*., 2021). To resolve this dilemma, plants employ compensatory mechanisms such as localized lignin biosynthesis, which simultaneously strengthens cell wall rigidity and reduces embolism propagation (Kong *et al*., 2023; Li *et al*., 2022). These adaptations enable perennial trees to maintain hydraulic functionality during water deficit while preserving biomechanical stability—a critical determinant of survival in intensifying drought regimes (Gessler *et al*., 2018; Zhang *et al*., 2022).

A critical threshold in drought‐induced xylem dysfunction is the percentage loss of diameter (PLD), which quantifies the cumulative hydraulic impairment through direct measurement of the diameter loss rate caused by drought stress (Andriantelomanana *et al*., 2024; Zhang *et al*., 2021). Unlike static anatomical metrics such as vessel density or mean vessel diameter, which reflect developmental traits under optimal conditions (Lens *et al*., 2011), the overall PLD measurement captures the dynamic interplay between structural collapse and physiological decline, providing a more robust predictor of drought‐induced mortality than static anatomical traits. Understanding the regulatory networks linking xylem adaptation to PLD dynamics is, therefore, essential for enhancing drought resistance and optimizing wood productivity under climate change.

*bZIP* transcription factors play critical roles in a wide range of biological processes, including regulation of plant growth and development (Bhatnagar *et al*., 2023; Zhao *et al*., 2021). In tobacco, the *bZIP* transcription factor subfamily I member *RSG* (*REPRESSION OF SHOOT GROWTH*) regulates gibberellin homeostasis by binding directly to the *NtGA20ox1* promoter, thereby exerting a negative feedback effect (Fukazawa *et al*., 2010; Nakata *et al*., 2009). Similarly, *SVF‐1* is expressed in vascular tissue and activates genes encoding structural cell wall proteins in tomato (Torres‐Schumann *et al*., 1996). In *Arabidopsis*, ectopic overexpression of *bZIP11* strongly inhibits plant growth in a concentration‐dependent manner (Ma *et al*., 2011). Additionally, *bZIP* transcription factors also play a role in drought stress (Guo *et al*., 2018). *OsMFT1* interacts with two key drought‐related transcription factors, *OsbZIP66* and *OsMYB26*, regulating their binding capacity to drought‐responsive genes and thereby enhancing drought tolerance (Chen *et al*., 2021). In *Arabidopsis*, IDD14 can physically interact with ABF1‐4 and subsequently promote their transcriptional activities (Liu *et al*., 2022b). In *Populus trichocarpa*, *PtrSnRK2.4* modulates drought tolerance and putrescine synthesis through *PtrABF2* phosphorylation (Song *et al*., 2022). Additionally, while these studies have underscored the significant roles of *bZIP* transcription factors in plant growth, development, and drought resistance, the molecular mechanisms linking xylem development to drought responses remain poorly understood.

In this study, we identified a PtoCIPK9*/PtoWRKY19–PtobZIP18–PtoCESA3/PtoGATL3/PtoDUF1635* module that coordinates stem growth and drought tolerance. Under drought stress, PtoCIPK9 destabilizes the PtobZIP18 protein through phosphorylation, while *PtoWRKY19* suppresses its transcription. This dual regulation downregulates *PtoGATL3* and *PtoCESA3*, which weaken the structural stability of vessel cells, and upregulates *PtoDUF1635*, which increases vessel diameter, collectively modulating poplar's water transport capacity under drought stress. Additionally, natural variation analysis revealed that low‐latitude accessions with reduced *PtobZIP18* expression exhibited enhanced drought resistance, whereas high‐latitude counterparts prioritized stem growth. Our findings represent a significant advance in understanding the regulatory network of stem adaptive development under drought stress, centred on *PtobZIP18*, serving as a foundation for precision breeding aimed at developing varieties with both high yield and enhanced drought resistance.

## Results

### *PtobZIP18* is a key candidate regulator involved in stem adaptive development under drought stress

PLD serves as a straightforward and effective indicator of stem responses to drought stress and is a critical metric for examining the effects of drought on stem adaptive development (Andriantelomanana *et al*., 2024). To explore phenotypic variation and the genetic basis of the PLD in a natural population of *Populus tomentosa*, a prolonged drought‐treatment trial was conducted with 300 juvenile *P. tomentosa* accessions in the field. The results revealed significant phenotypic differentiation in PLD among three subpopulations (south [S], northeast [NE] and northwest [NW]), which is consistent with three geographical climatic regions (Li *et al*., 2023). Specifically, accessions from higher latitudes (the NE and NW populations) exhibited lower PLD compared to those from lower latitudes (the S population) (Figure S1a), which may have resulted from genetic adaptation to local climatic and geographical conditions.

**Figure 1:**
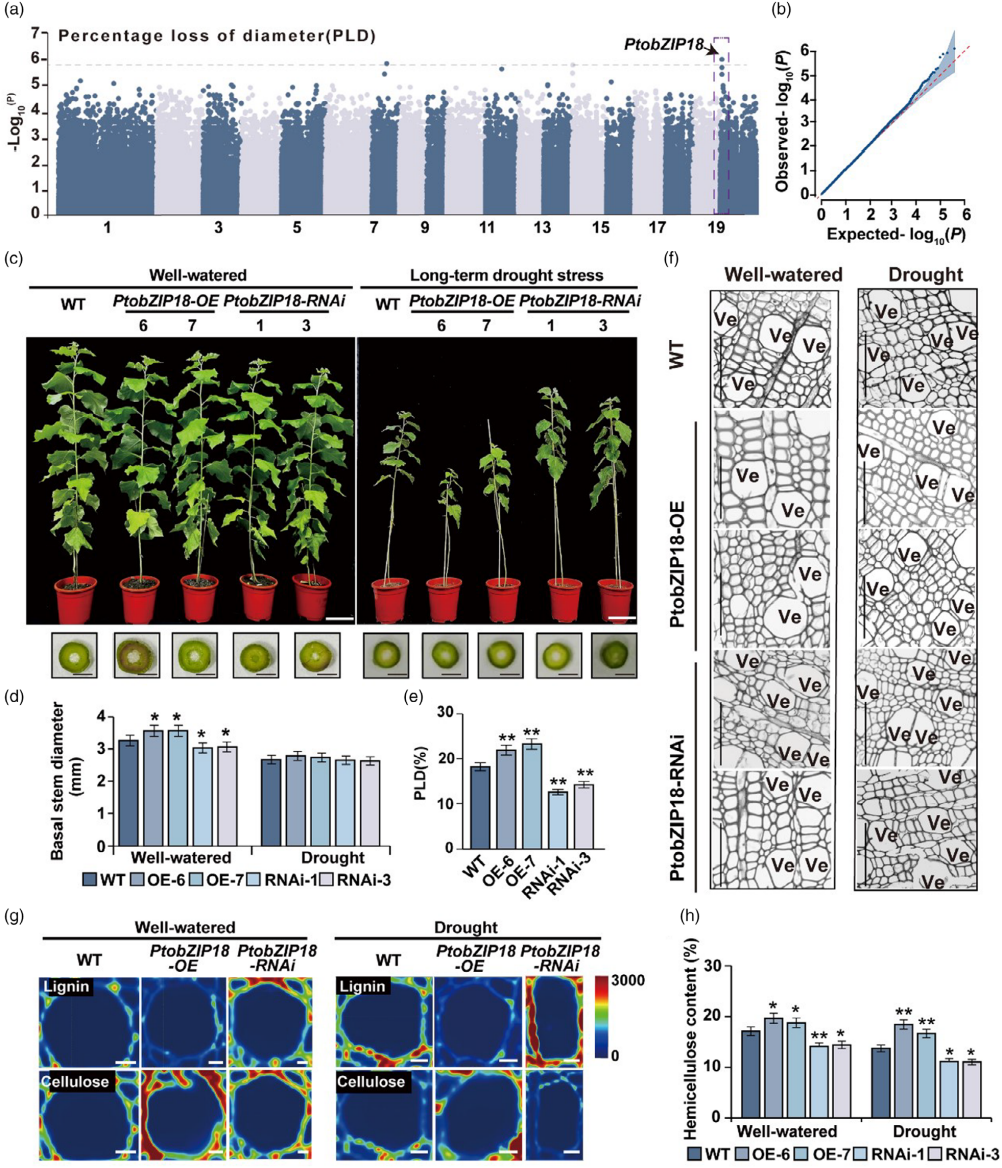
Morphology and physiology characteristics of wild‐type (WT), *PtobZIP18‐OE*, and *PtobZIP18‐RNAi* transgenic poplars under normal and drought conditions. (a) Manhattan plot of GWAS for percentage loss of diameter after adequate water growth and drought treatment. P‐values are calculated based on a linear mixed model in GWAS, and the dashed horizontal line indicates the genome‐wide significance threshold (*P* = 1.75 × 10^−6^), which is determined by the Bonferroni test. (b) QQ plots of the GWAS. (c) The phenotype analysis of plants. Bars, 10 cm (above). Stem cross‐section of transgenic plants and WT. Bars, 2 mm (bottom). Three individual poplars for each genotype were used, and each showed similar results, with a representative picture shown. (d) Basal stem diameter of plants. Each mean and standard deviation were calculated from at least 3 plants. Values represent the mean ± SD of three biological replicates. Student's t‐tests: Student's *t*‐test: *P < 0.05; **P < 0.01. (e) PLD trait in WT, *PtobZIP18*‐OE, and *PtobZIP18‐RNAi* plants was measured after 40 days of drought stress. (f) Cytological observations of stem cross sections of 15th internodes. Bars, 40 μm. Ve, vessel. (g) CRM images of vessels, Scale bars, 10 μm. (h) hemicellulose content of plants under well‐watered condition and drought‐stress condition. Three independent experiments were performed. Statistical analysis was performed with Student's *t*‐test (*P < 0.05, **P < 0.01), data are provided as means ± SDs.

To elucidate the genetic basis of stem development associated with drought tolerance, we performed a genome‐wide association study (GWAS) of PLD traits, which identified six significant single‐nucleotide polymorphisms (SNPs) (*P* < 1.75 × 10^−6^; 1/*n*, Bonferroni test). These SNPs were annotated to six candidate genes (Figure 1a,b). Among the candidate genes, the expression of *Ptom.019G.00085* was significantly higher in cambium, immature xylem, and mature xylem tissues, whereas the other genes lacked tissue‐specific expression (Figure S1b,c). Additionally, *Ptom.019G.00085* was notably downregulated under drought stress (Figure S1d). Based on these findings, we selected *Ptom.019G.00085* as a key candidate gene regulating PLD traits. Given its possession of a basic leucine zipper (bZIP) domain and its high homology with *AtbZIP18* in *Arabidopsis*, we designated it *PtobZIP18* (Figure S1e).

### *PtobZIP18* modulates stem growth and drought response through xylem regulation

To further elucidate the function of *PtobZIP18* in growth–drought trade‐offs, we generated *PtobZIP18* overexpression (*PtobZIP18‐OE*) and knockdown (*PtobZIP18‐RNAi*) lines (Figure S2a). Under well‐watered conditions, *PtobZIP18‐OE* lines exhibited increased basal stem diameters, while *PtobZIP18‐RNAi* lines showed reduced diameters compared to WT (Figure 1c,d), establishing its growth‐promoting function in well‐watered conditions.

To more intuitively confirm the role of *PtobZIP18* in drought sensitivity, we conducted a short‐term drought for 10 days. *PtobZIP18‐RNAi* lines accumulated higher free proline but lower H_2_O_2_ and malondialdehyde (MDA) levels than WT, whereas *PtobZIP18‐OE* lines showed the opposite trends (Figure S2b–e). These results suggest that *PtobZIP18* negatively regulates drought tolerance in poplar. Strikingly, prolonged drought (40 days at 25% soil moisture) erased the transgenic poplars growth differences (Figure 1c,d), with *PtobZIP18‐OE* suffering 4.4% higher PLD and *PtobZIP18‐RNAi* lines maintaining 4.9% lower PLD compared to WT (Figure 1e), suggesting a survival cost for *PtobZIP18*‐driven growth. These results imply that *PtobZIP18*‐mediated stem growth promotion is compromised under drought due to its intrinsic role in suppressing drought adaptation.

To further investigate the cause of the growth–drought trade‐off, the stem xylem structure was analysed. Under well‐watered conditions, there was an 8.0% reduction in vessel density and a 17.8% increase in vessel lumen area in *PtobZIP18‐OE* plants compared to WT. Whereas *PtobZIP18‐RNAi* plants showed a 31.2% increase in vessel density and a 12.3% reduction in vessel lumen area. Under drought stress, *PtobZIP18‐OE* plants displayed a further 4.9% decrease in vessel density and a 15.1% increase in lumen area. While *PtobZIP18‐RNAi* plants had a 6.7% increase in vessel density and an 11.9% decrease in vessel lumen area (Figure 1f; Figure S2f,g). These results suggest that changes in the vessel structure of *PtobZIP18‐OE* impair drought resistance, and drought‐induced xylem remodelling enhances drought adaptation. Concomitant changes in wood composition paralleled the structural adaptations. Under both normal and drought conditions, lignin content was significantly reduced in *PtobZIP18‐OE* plants, accompanied by elevated levels of cellulose and hemicellulose, whereas *PtobZIP18‐RNAi* plants exhibited inverse changes (Figure 1g,h; Figure S2h–k). Notably, the differences in wood composition between transgenic and WT plants under drought stress were less pronounced under drought stress than under well‐watered conditions (Figure 1g,h; Figure S2h–k). Consistent with the xylem structural alterations, stem water conductivity, stem water potential, and relative water content (RWC) were significantly reduced in the *PtobZIP18‐OE* plants, whereas significantly increased in the *PtobZIP18‐RNAi* plants compared to WT under drought conditions (Figure S2l–n). These findings indicated that the alteration of the xylem structure in *PtobZIP18‐OE* plants reduced their water transport capacity, leading to reduced drought tolerance.

### Natural variation in *PtobZIP18* promoter is related to environmental adaptation

**Figure 2:**
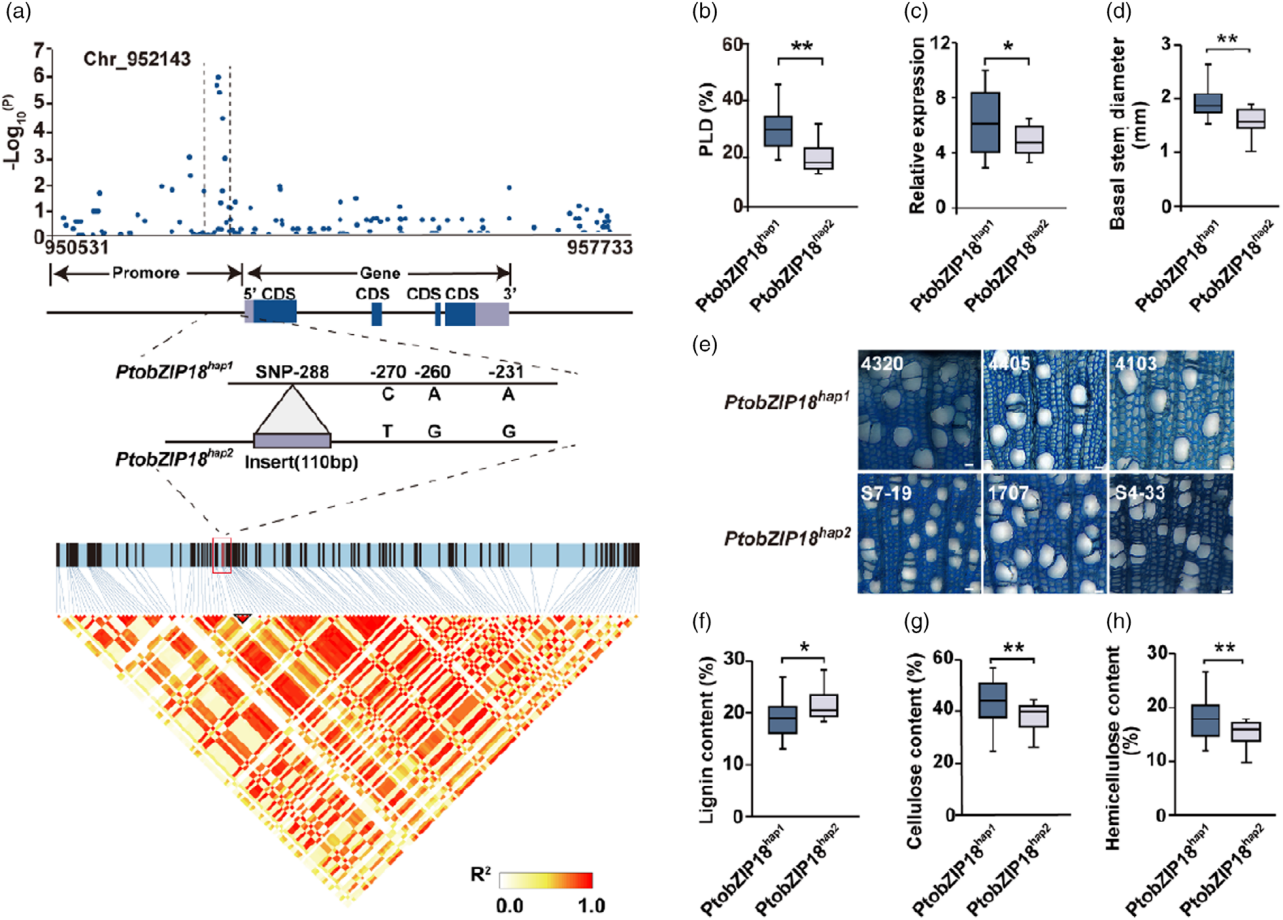
Natural variation in *PtobZIP18* confers the trade‐off between stem growth and drought tolerance in *Populus*. (a) Association analysis of genetic variation in *PtobZIP18* with the PLD (top), haplotypes of *PtobZIP18* among *Populus* natural variations (middle) and linkage disequilibrium heat map (bottom) for the *PtobZIP18* gene, which is associated with PLD. *PtobZIP18* exhibits natural variation in poplar, forming two distinct haplotypes characterized by one SV and three SNP mutations. (b) Comparison of the PLD trait between *PtobZIP18* ^*hap1*^ and *PtobZIP18* ^*hap2*^ in poplar. (c) Comparison of the expression level of *PtobZIP18* between *PtobZIP18* ^*hap1*^ and *PtobZIP18* ^*hap2*^ in poplar. (d) Basal stem diameter of *PtobZIP18* ^*hap1*^ and *PtobZIP18* ^*hap2*^. (e) Stem cross‐section of six accessions of *P. tomentosa* stained with toluidine blue. The six accessions were divided into *PtobZIP18* ^*hap1*^ and *PtobZIP18* ^*hap2*^ based on the natural alleles of *PtobZIP18*. Scale bars represent 50 μm, respectively. (f–h) Lignin (f), cellulose (g) and hemicellulose content (h) of *PtobZIP18* ^*hap1*^ and *PtobZIP18* ^*hap2*^. Asterisks show significant differences by Student's *t*‐test: *P < 0.05; **P < 0.01.

To elucidate the natural variation within *PtobZIP18*, a 7.2‐kb region encompassing the full‐length coding sequence (CDS) and 2‐kb upstream regulatory region was selected across the re‐sequenced natural population of 300 accessions. Structural variation (SV) analysis of this SNPs‐associated genomic region revealed a major haplotype block, comprising one SV and three SNPs, in the *PtobZIP18* promoter that divided the 300 accessions into two distinct haplotypes: *PtobZIP18* ^*hap1*^ and *PtobZIP18* ^*hap2*^ (Figure 2a; Data S1), appearing to underlie the phenotypic variations in PLD under drought stress observed across different populations. Accessions carrying the *PtobZIP18* ^*hap1*^ (118 accessions) were associated with relatively high PLD values, whereas those carrying the *PtobZIP18* ^*hap2*^ (34 accessions) were associated with relatively low PLD (Figure 2b). Gene expression assays confirmed that *PtobZIP18* ^*hap1*^ had significantly higher expression levels than *PtobZIP18* ^*hap2*^ (Figure 2c), indicating that sequence variations between haplotypes drive differential *PtobZIP18* expression.

We subsequently evaluated whether the natural variation of *PtobZIP18* alters the stem growth and drought tolerance of natural *P. tomentosa* varieties. The accessions carrying the *PtobZIP18* ^*hap1*^ have a larger basal stem compared to those carrying the *PtobZIP18* ^*hap2*^ under well‐watered conditions, indicating that the stems of accessions carrying the *PtobZIP18* ^*hap1*^ exhibit stronger growth ability (Figure 2d). Furthermore, we performed paraffin sectioning, which demonstrated that plants carrying the *PtobZIP18* ^*hap2*^ had higher vessel densities and smaller vessel diameters compared to those with the *PtobZIP18* ^*hap1*^ (Figure 2e; Figure S3a,b). Additionally, *PtobZIP18* ^*hap2*^ plants had reduced cellulose and hemicellulose contents, coupled with increased lignin contents (Figure 2f–h). These structural and compositional changes provide compelling evidence for enhanced drought resistance being associated with the *PtobZIP18* ^*hap2*^.

The geographic distribution of haplotypes provided evolutionary context for these phenotypic tradeoffs. Further investigation of *PtobZIP18* haplotypes across the 300 re‐sequenced accessions revealed *PtobZIP18* ^*hap1*^ occurred in all three climatic regions, with the highest frequency in the S climate region, while *PtobZIP18* ^*hap2*^ was primarily distributed in the NW climate region but absent in the S climate region (Figure S3c). These findings imply that haplotype may be related to the adaptation of plants to specific climatic conditions.

### Allelic variation in the *PtobZIP18* promoter affects the binding affinity of its upstream regulator PtoWRKY19

**Figure 3:**
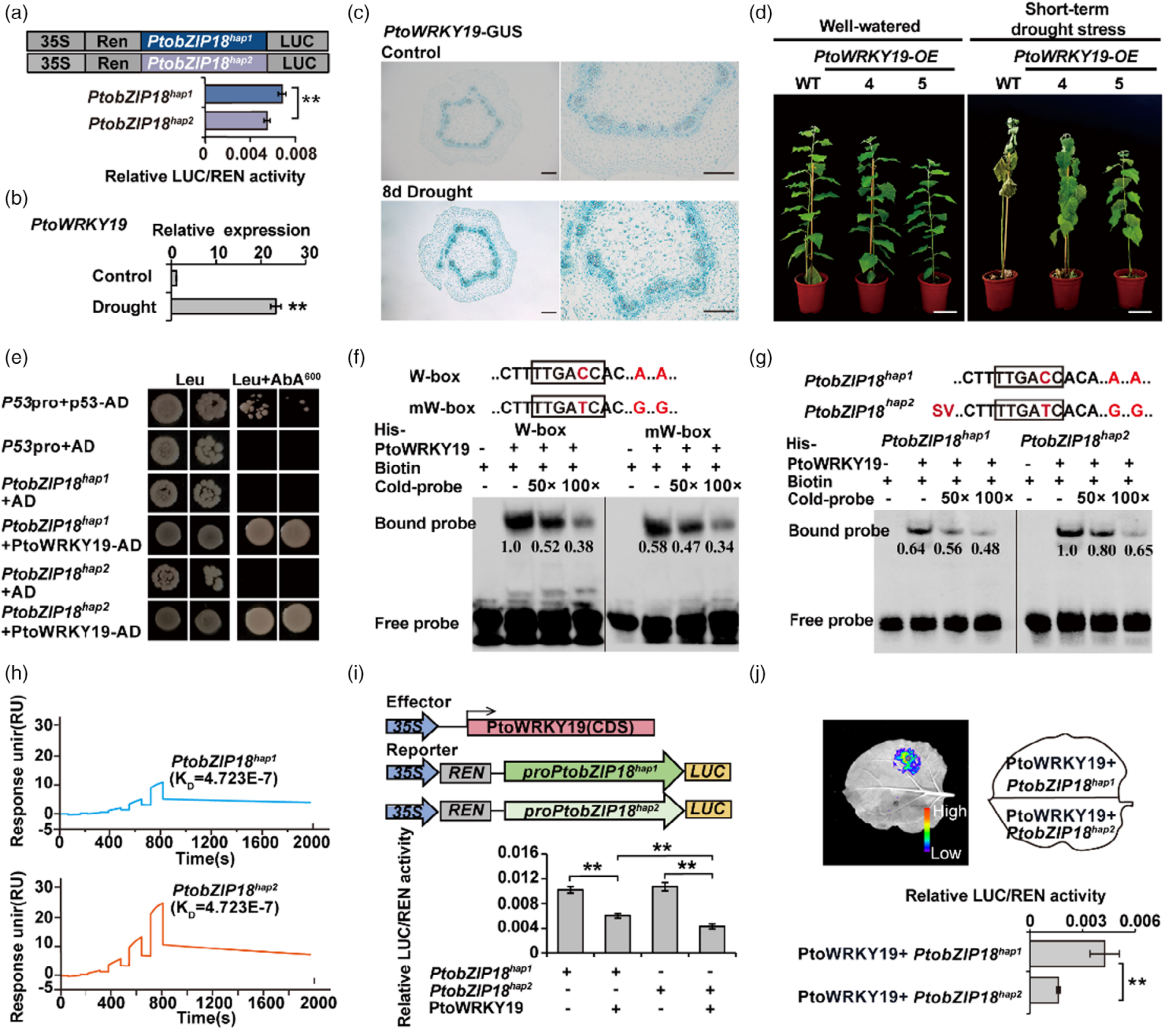
*PtoWRKY19* directly binds the promoters of *PtobZIP18* ^*hap1*^ and *PtobZIP18* ^*hap2*^ to inhibit their transcription. (a) Promoter activity analysis of *PtobZIP18* ^*hap1*^ and *PtobZIP18* ^*hap2*^ using sequences 2000‐bp upstream from the translation initiation site (*n* = 4 biologically independent replicates). (b) Relative expression level of *PtoWRKY19* was determined by RT‐qPCR in poplar with sufficient water and 40 days under drought stress, respectively. The poplar Actin was used as an internal standard for data normalization. Values are means ± SD. *n* = 3. (c) GUS histochemical staining of the stems of 1‐month‐old soil‐cultivated *proPtoWRKY19*: Gus transgenic lines under control or 8 d drought treatment. Bars: 200 μm. (d) Growth phenotypes of 3‐month‐old *PtoWRKY19‐OE* and WT plants. Bar, 10 cm. (e) Yeast one‐hybrid assay showing that *PtoWRKY19* binds to *PtobZIP18* ^*hap1*^ and *PtobZIP18* ^*hap2*^ promoters. AD‐Rec‐P53 and P53‐promoter‐AUR1‐C were used as the positive controls, AD‐Empty prey vector and P53‐promoter‐AUR1‐C were used as the negative controls. Aureobasidin A (AbA), a yeast cell growth inhibitor, was used as a screening marker. (f) Electrophoretic mobility shift assay (EMSA) of PtoWRKY19 binding to *W‐box* and *mW‐box* promoter. (g) Electrophoretic mobility shift assay (EMSA) of PtoWRKY19 binding to *PtobZIP18* ^*hap1*^ and *PtobZIP18* ^*hap2*^ promoter. In (f, g), the biotin‐labelled probe serves as a negative control in Lane 1, while Lane 2 contains the biotin‐labelled probe incubated with PtoWRKY19‐HIS protein. Lanes 3 and 4 demonstrate competitive binding with 50× and 100× molar excess of unlabeled probe, respectively. Three independent experiments yielded consistent results (representative image shown). (h) SPR binding profiles of PtoWRKY19 proteins to the promoter of *PtobZIP18* ^*hap1*^ and *PtobZIP18* ^*hap2*^. RU, response units; KD, dissociation constant. Single‐cycle kinetic analysis of intermolecular interactions using OC at concentrations of 0.5363, 1.341, 3.352, 8.38, and 20.95 μM, assessed via SPR‐based assay. (i, j) Relative luciferase activity according to a DLRA assay of *N. benthamiana* leaves. Quantification was performed by normalizing firefly luciferase (LUC) activity to that of Renilla luciferase (REN), *35S*: REN was used as the internal control. The values represent the mean ± SD standard deviation of the three biological replicates. Student's *t*‐test: *P < 0.05; **P < 0.01.

To determine whether natural variation in the *PtobZIP18* promoter contributes to differences in its expression levels, we performed a transient transcription activity assay. This assay showed that the *PtobZIP18* ^*hap1*^ had significantly higher transcriptional activity than the *PtobZIP18* ^*hap2*^ promoter (Figure 3a). These results indicated that the differences in transcriptional activity between *PtobZIP18* haplotypes arise from variations in their promoter regions. Mutation sequence analysis of the *PtobZIP18* promoter in both haplotypes revealed that no known conserved transcription factor binding motifs were detected in the SV region. However, we identified that one significantly associated SNP altered the *WRKY*‐binding W‐box element (Pandey and Somssich, 2009), changing its core motif from “TGAC” to “TGAT” (Figure S4a). This finding prompted us to investigate potential interacting WRKY transcription factors. Based on transcriptome data from long‐term drought‐stress experiments, tissue‐specific expression analysis, and correlation analysis, we identified a gene named *PtoWRKY19* (Figure S4b–d, Data S2). RT‐qPCR and GUS staining confirmed xylem‐specific expression of *PtoWRKY19*, with significant induction in stems under drought stress (Figure 3b,c, Figure S4e). These findings imply that *PtoWRKY19* primarily functions in poplar stems under water‐deficient conditions. *PtoWRKY19‐OE* lines were generated and validated by RT‐qPCR (Figure 3d; Figure S4f,g). Under short‐term drought, *PtoWRKY19‐OE* plants exhibited higher free proline but lower H_2_O_2_ and MDA levels than WT (Figure S4h–j). This enhanced stress tolerance translated to an 87% survival rate in *PtoWRKY19‐OE* versus 68% in WT (Figure S4k), confirming *PtoWRKY19* as a positive regulator of drought tolerance.

To investigate whether PtoWRKY19 regulates *PtobZIP18*, we evaluated *PtobZIP18* expression with RT‐qPCR. Our results showed that *PtobZIP18* was significantly downregulated in *PtoWRKY19‐OE* plants (Figure S4l). To investigate whether *PtobZIP18* is a direct target of PtoWRKY19, we performed yeast one‐hybrid (Y1H) assays, which confirmed that PtoWRKY19 bound directly to both *PtobZIP18* ^*hap1*^ and *PtobZIP18* ^*hap2*^ (Figure 3e). Electrophoretic mobility shift assays (EMSAs) further demonstrated that mutation of the W‐box alone reduced the binding affinity to PtoWRKY19. Intriguingly, while probes corresponding to both haplotypes retained the ability to bind PtoWRKY19, the *PtobZIP18* ^*hap2*^ probe carrying mutations in both the W‐box and SV elements exhibited significantly stronger binding (Figure 3f,g). Surface plasmon resonance (SPR) analysis corroborated these findings, indicating that PtoWRKY19 had a higher binding affinity for *PtobZIP18* ^*hap1*^ compared to *PtobZIP18* ^*hap2*^ (Figure 3h). Luciferase complementation imaging (LCI) experiments revealed that *PtoWRKY19* suppressed the transcriptional activity of *PtobZIP18*, with stronger inhibitory effects in *PtobZIP18* ^*hap2*^ (Figure 3i,j; Figure S4m). In conclusion, the dual effects of SNPs and SV resulted in PtoWRKY19 having stronger inhibition of *PtobZIP18* ^*hap2*^.

### PtobZIP18 directly activates the expression of *PtoGATL3* , *PtoCESA3* and *PtoDUF1635*

The C‐terminus of PtobZIP18 protein contains a bZIP domain, comprising a basic DNA‐binding region and a leucine zipper domain (E *et al*., 2014; Figure S5a). Consistent with its role as a bZIP transcription factor, the PtobZIP18‐GFP fusion protein was observed to localize to the nucleus (Figure S5b). Additionally, transcriptional activity analysis in yeast showed that PtobZIP18 functions as a transcriptional activator (Figure S5c).

**Figure 4:**
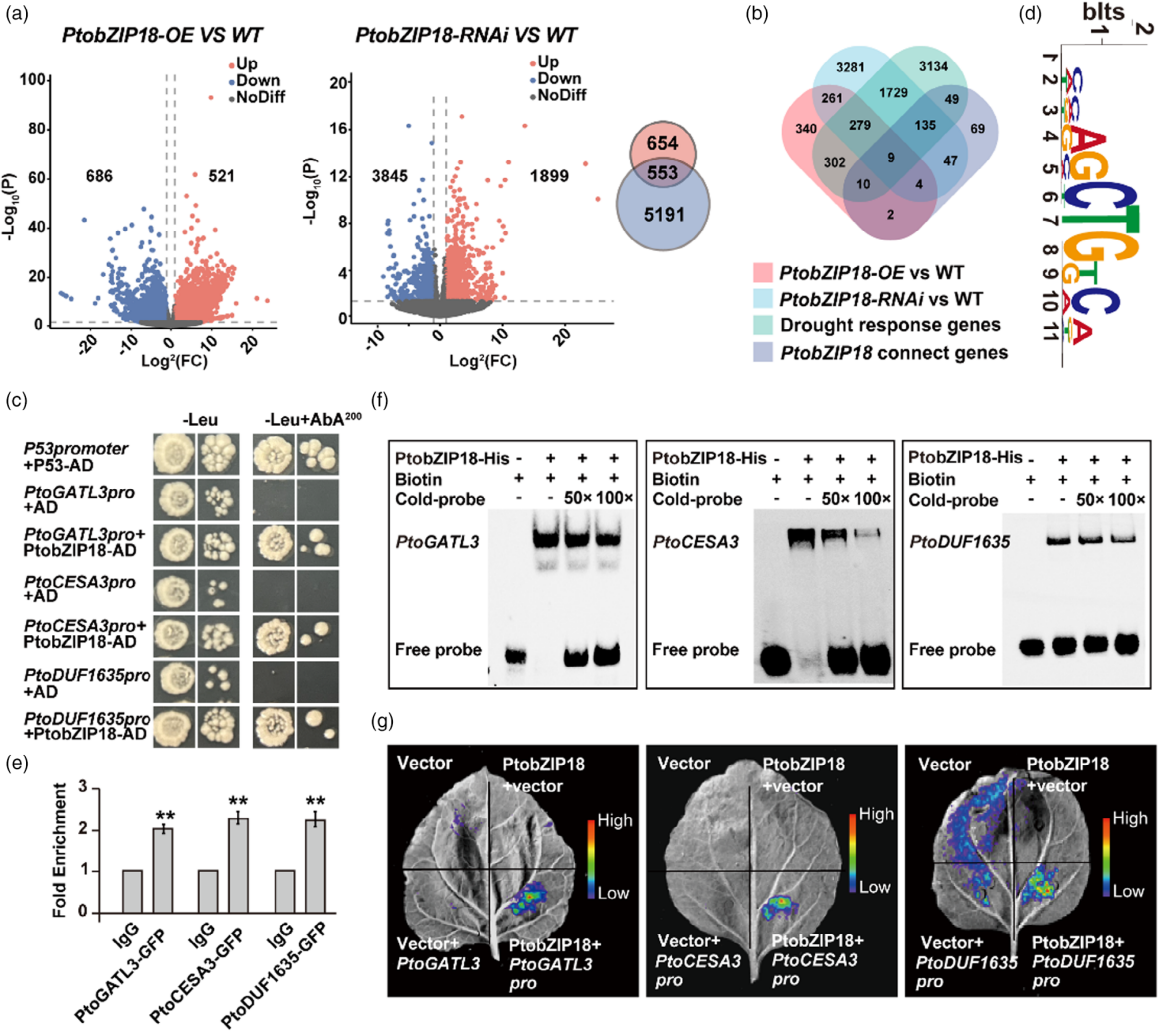
*PtobZIP18* directly binds the promoter of *PtoCESA3, PtoGATL3, PtoDUF1635* and positively regulates their expression. (a) Volcano plots representing the fold‐change of DEGs in the comparison groups of WT versus *PtobZIP18‐OE*, WT versus *PtobZIP18‐RNAi* (*P* < 0.05, absolute fold‐change ≥2.0). Grey dots represent genes without significant changes in expression. Three independent experiments were performed for each sample at each time point. (b) Venn diagram showing the DEGs that jointly exist in four terms. (c) Yeast one‐hybrid assay showing that PtobZIP18 binds to *PtoCESA3, PtoGATL3* and *PtoDUF1635* promoters. AD‐Rec‐P53 and P53‐promoter‐AUR1‐C were used as the positive controls; AD‐Empty prey vector and AUR1‐C under the control of one target gene promoter vector were used as the negative controls. Aureobasidin A (AbA), a yeast cell growth inhibitor, was used as a screening marker. (d) Motif analysis of bZIP18‐binding regions comes from Plant TFDB published data. (e) ChIP‐qPCR assay revealed the binding of PtobZIP18 protein fused with the GFP tag to the promoter region of *PtoGATL3*, *PtoCESA3* and *PtoDUF1635*. (f) Electrophoretic mobility shift assay (EMSA) of PtobZIP18 binding to *PtoCESA3, PtoGATL3* and *PtoDUF1635* promoters. Biotin‐labelled probe was used as a negative control (Lane 1). Biotin‐labelled probe incubated with PtobZIP18‐HIS protein was tested (Lane 2). Competitive probes of 50× and 100× (lack of biotin label) were used (Lanes 3 and 4). Three independent experiments were performed with a representative picture shown. (g) Transient dual‐luciferase (dual LUC) assays of PtobZIP18 binding to the promoters of *PtoGATL3, PtoCESA3* and *PtoDUF1635* in tobacco leaves.

To investigate the molecular mechanisms of *PtobZIP18* in regulating xylem structure and water transport capacity under drought stress, we analysed transcriptomes of *PtobZIP18‐OE* and *PtobZIP18‐RNAi* lines under stress, identifying 553 common differentially expressed genes (DEGs) (Figure 4a; Figure S5d; Data S3). We further analysed the differential expression of xylem transcriptomes following 40 days of “LM50” drought treatment (Data S4). Concurrently, we performed weighted gene co‐expression network analysis (WGCNA) using xylem transcriptomic data from *Populus* under drought stress (available from NCBI) to identify *PtobZIP18*‐centred interaction modules (Data S5). Integration of four independent datasets revealed nine genes as putative targets of PtobZIP18 (Figure 4b; Data S6).

**Figure 5:**
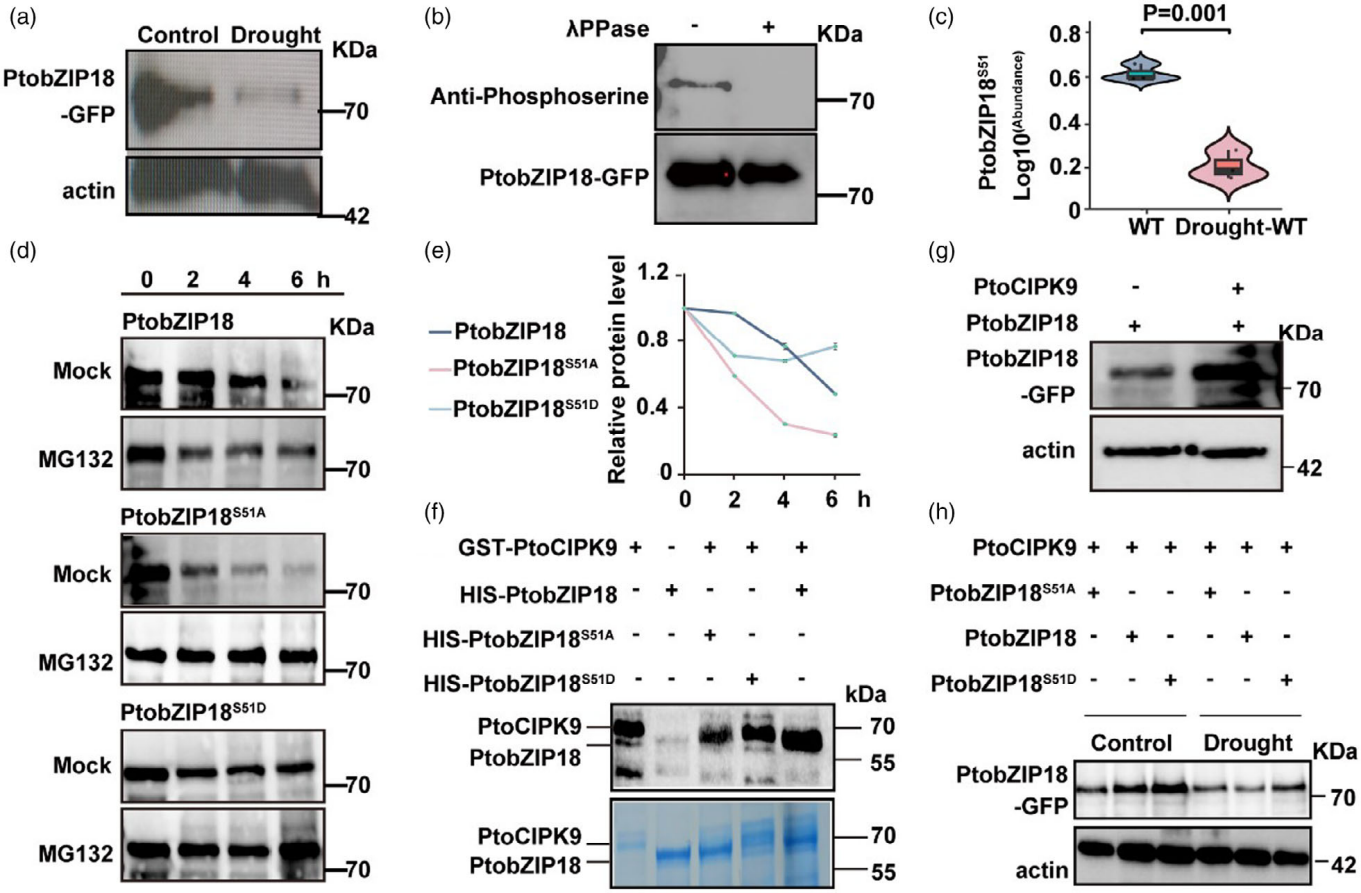
PtoCIPK9‐Mediated phosphorylation of PtobZIP18 decreases drought tolerance. (a) PtobZIP18 protein abundance of two‐month‐old *Populus* grown under well‐watered or drought treated conditions for 40 days. PtobZIP18 protein was detected with anti‐GFP antibody. PtobZIP18 protein levels were normalized to Actin. (b) PtobZIP18 is phosphorylated at serine (Ser) *in vivo*. Plant total proteins of PtobZIP18‐GFP overexpressing plants were extracted and subjected to digestion with λ‐PPase at 30 °C for 30 min. The samples were then subjected to immunoblot analysis and detected with anti‐GFP and anti‐phosphoserine antibodies. (c) Phosphorylation activity of PtobZIP18 at ser 51 position under normal and drought stress. (d) Recombinant proteins of PtobZIP18, PtobZIP18^S51A^ and PtobZIP18^S51D^ were expressed and purified from *Escherichia coli*. Recombinant proteins were incubated with or without 50 mM MG132 (a proteasome inhibitor) at 37 °C in cell lysate from the xylem of *Populus* grown at the 3‐month‐old stage. PtobZIP18, PtobZIP18^S51A^ and PtobZIP18^S51D^ accumulation were examined by immunoblotting assay. (e) Protein abundance of (d) was quantified based on the signal density from three replicates. (f) PtoCIPK9 phosphorylates PtobZIP18 *in vitro*. Recombinant HIS‐PtobZIP18, HIS‐PtobZIP18^S51A^, HIS‐PtobZIP18^S51D^ and GST‐PtoCIPK9 proteins were subjected to an *in vitro* phosphorylation assay. The autoradiogram (top) and the Coomassie Brilliant Blue‐stained gel (bottom) are shown. (g) PtobZIP18 protein abundance in poplar protoplasts. PtobZIP18 protein levels were normalized to Actin. (h) PtobZIP18 protein abundance in *N. benthamiana* leaf cells under normal or drought‐treated conditions for 2 h. PtobZIP18 protein was detected with anti‐GFP antibody. PtobZIP18 protein levels were normalized to Actin.

Y1H assays revealed that PtobZIP18 specifically binds to the promoters of *PtoGATL3, PtoCESA3* and *PtoDUF1635* (Figure 4c, Figure S5e). Under drought, these genes were downregulated in WT but upregulated in *PtobZIP18‐OE* lines (Figure S5f,g). Expression profiles showed high levels of *PtoGATL3*, *PtoCESA3* and *PtoDUF1635* in xylem, similar to *PtobZIP18* (Figure S5h–j). Chromatin immunoprecipitation (ChIP)‐qPCR, EMSAs and Luciferase complementation imaging (LCI) confirmed PtobZIP18 binding to the “HNRVCTGKMN” motif in their promoters (https://planttfdb.gao‐lab.org/) (O'Malley *et al*., 2016) and PtobZIP18 activates their transcription (Figure 5e–g). These results demonstrate that PtobZIP18 directly binds to and activates *PtoCESA3*, *PtoGATL3* and *PtoDUF1635*.

### Drought destabilizes PtobZIP18 by attenuating PtoCIPK9‐mediated phosphorylation

Immunoblot analysis revealed drought‐induced degradation of PtobZIP18‐GFP in stem tissues (Figure 5a). Given the established link between bZIP phosphorylation and protein stability (Li *et al*., 2022; Shi *et al*., 2024; Sirichandra *et al*., 2010), we performed anti‐phosphotyrosine immunoprecipitation to examine whether the drought‐induced decrease in PtobZIP18 abundance is due to phosphorylation. λ‐PPase treatment abolished the phosphorylation of PtobZIP18‐GFP (~71 kDa) (Figure 5b), confirming its phosphorylated state. To explore further whether changes in PtobZIP18 protein levels were related to its phosphorylation status, we analysed phosphorylation activity under normal and drought conditions. Drought specifically reduced phosphorylation at Ser51 (Figure 5c), suggesting a drought‐induced reduction in PtobZIP18 phosphorylation level may contribute to its decreased protein levels.

To further examine the role of phosphorylation in PtobZIP18 protein stability, we generated two mutant PtobZIP18 proteins in which Ser51 was substituted with Ala (PtobZIP18^S51A^) and Asp (PtobZIP18^S51D^). Recombinant PtobZIP18, PtobZIP18^S51A^ and PtobZIP18^S51D^ proteins were expressed in *E. coli*, and their stability was evaluated in cell lysates from developing xylem of *Populus* plants. Additionally, treatment with the 26S proteasome inhibitor MG132 prevented the degradation of His‐PtobZIP18 (Figure 5d,e). These findings indicated that phosphorylation enhances the stability of the PtobZIP18 protein.

To identify protein kinases that phosphorylate PtobZIP18, we performed yeast two‐hybrid (Y2H) assays. The results suggested direct interactions between PtobZIP18 and several protein kinases, with a particularly notable correlation between the expression levels of PtobZIP18 and PtoCIPK9 (CBL‐INTERACTING PROTEIN KINASE 9) (Figure S5a, Data S8). Notably, PtoCIPK9 participated in drought tolerance via ABA signalling in *Arabidopsis* (Song *et al*., 2018). The interaction between PtoCIPK9 and PtobZIP18 was confirmed both *in vitro* and *in vivo* through one‐to‐one Y2H and split luciferase (split‐LUC) complementation assays (Figure S5b,c). Additionally, liquid chromatography–tandem mass spectrometry (LC–MS/MS) of recombined proteins confirmed that PtoCIPK9 phosphorylated PtobZIP18‐GFP at the Ser51 residue (Figure S5d). *In vitro* kinase assays demonstrated that PtoCIPK9 phosphorylated PtobZIP18; however, no phosphorylation signal was detected when Ser51 was mutated to Ala (Figure 5f). These results indicate that PtoCIPK9 phosphorylates PtobZIP18 at Ser51 *in vitro*. To validate these findings, we performed western blot analysis in PtoCIPK9/PtobZIP18‐coexpressing poplar protoplasts, which showed that PtoCIPK9 enhances the stability of PtobZIP18‐GFP (71.2 kDa) (Figure 5g). To further investigate the functional consequences of phosphorylation, we conducted dual‐luciferase reporter assays in tobacco leaves. The results showed that PtoCIPK9‐mediated phosphorylation had no significant effect on its transcriptional activation capacity (Figure S6e). Furthermore, in the tobacco system, drought stress reduced PtobZIP18 protein levels within 2 h (Figure 5h). These results demonstrate that drought disrupts PtoCIPK9‐mediated phosphorylation, leading to PtobZIP18 destabilization.

### *PtoGATL3* and *PtoCESA3* promote cellulose and hemicellulose biosynthesis and are negatively correlated with drought tolerance

**Figure 6:**
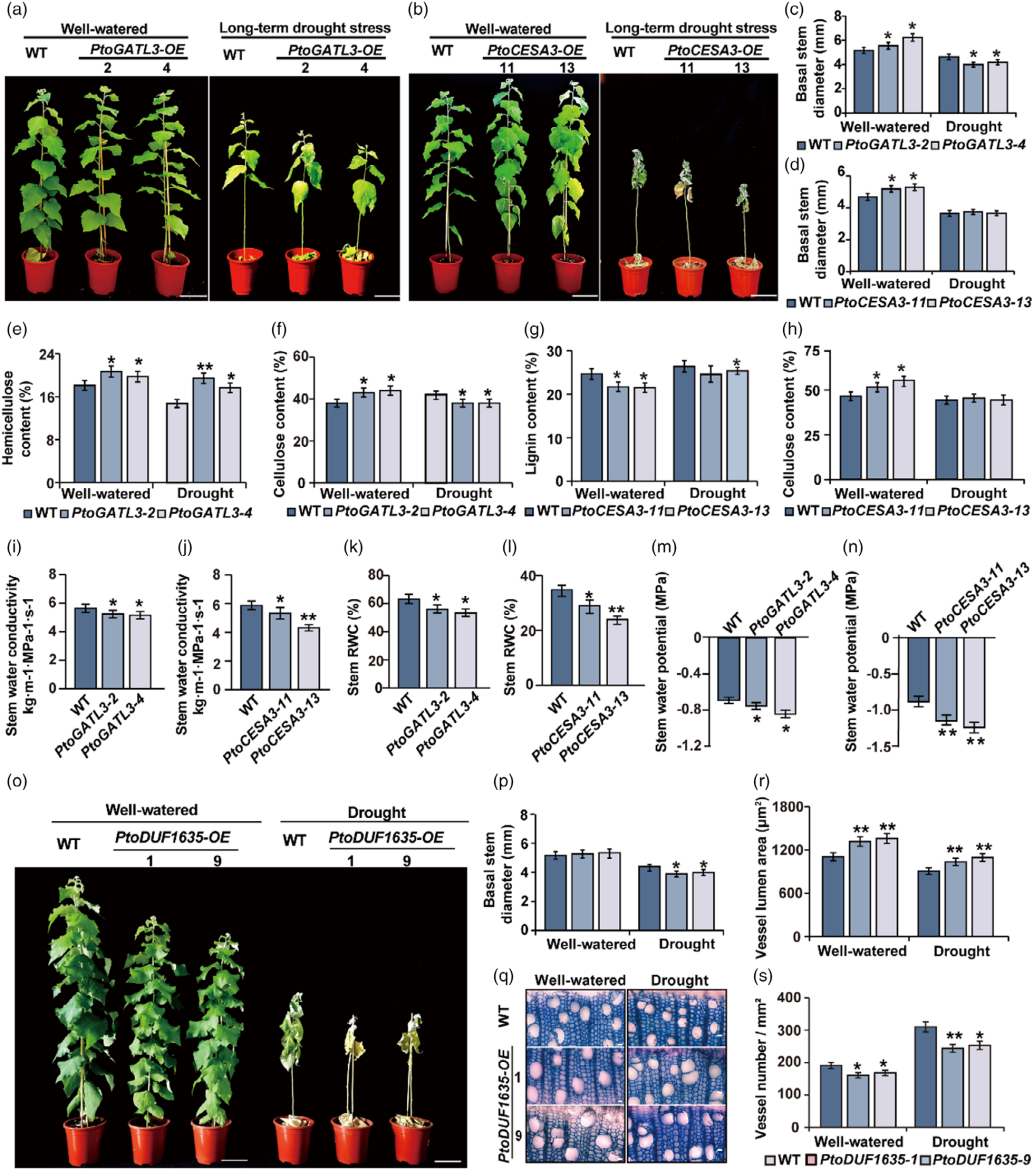
Morphology and physiology characteristics of wild‐type (WT), *PtoGATL3‐OE*, *PtoCESA3‐OE* and *PtoDUF1635‐OE* transgenic poplars under normal and drought conditions. The 4‐week‐old tissue‐cultured *Populus* plantlets were planted in soil and maintained for a period of 50 days under well‐watered conditions. Subsequently, the plants were either grown under well‐watered conditions for 40 days as a control, and the soil water content was carefully regulated and kept within the range of 20–25% for 40 days as drought conditions. (a, b) Growth phenotypes of *PtoGATL3*‐*OE* A) and *PtoCESA3‐OE* B) poplars. Scale bar, 10 cm. (c, d) Basal stem diameter of *PtoGATL3‐OE* (c) and *PtoCESA3‐OE* (d) poplars. (e, f) Hemicellulose content (e) and cellulose content (f) of analysed in the WT and *PtoGATL3‐OE* stem. (g, h) Lignin content (g) and cellulose content (h) of analysed in the WT and *PtoCESA3‐OE* stem. (i–n) Stem water conductivity (i, j), stem relative water content (k, l), and Stem water conductivity (m, n) of plants under drought‐stress condition. Three independent experiments were performed. Statistical analysis was performed with Student's *t*‐test: *P < 0.05; **P < 0.01; data are provided as means ± SDs. (o) Growth phenotypes of WT and *PtoDUF1635*‐*OE* under normal and drought conditions. Scale bar, 10 cm. (p) Vessel morphology observations of stem cross sections of 15th internodes. Bar, 50 μm. (r, s) vessel lumen area (r), and vessel number (s) from 3‐month‐old *PtoDUF1635‐OE and* WT under well‐watered condition and drought condition. Values represent the mean ± SD of three biological replicates. Student's *t*‐test: *P < 0.05; **P < 0.01.

Overexpression lines of *PtoGATL3* and *PtoCESA3* exhibited increased basal stem diameters compared to WT under well‐watered conditions (Figure S7a,b; Figure 6a–d). Cell wall composition analysis revealed that *PtoGATL3‐OE* lines accumulated higher hemicellulose and cellulose levels without altered lignin content, whereas *PtoCESA3‐OE* lines showed elevated cellulose but reduced lignin content (Figure 6e–h; Figure S7c,d), consistent with known roles of their *Arabidopsis* homologues in xylose metabolism and cellulose synthesis (Kong *et al*., 2011; Rogers *et al*., 2005; Xin *et al*., 2023). Vessel structure remained unchanged in transgenic lines (Figure S7e–g). These results demonstrate that *PtoGATL3* and *PtoCESA3* specifically coordinate cellulose and hemicellulose accumulation while suppressing lignin biosynthesis.

Under short‐term drought, *PtoGATL3‐OE* and *PtoCESA3‐OE* lines exhibited lower free proline but higher H_2_O_2_ and MDA levels than WT (Figure S7h–l), indicating increased drought vulnerability. Under prolonged drought, *PtoGATL3‐OE* lines showed reduced basal stem diameters compared to WT, while *PtoCESA3‐OE* lines were similar to WT (Figure 6a–d). Both transgenic lines exhibited higher PLD values, indicating greater drought sensitivity (Figure S7m). Cellulose content decreased in *PtoGATL3‐OE* lines, while lignin content was reduced in *PtoCESA3‐OE* lines (Figure 6e–h). Stem water conductivity, water potential, and RWC were significantly lower in transgenic lines than in WT (Figure 6i–n), suggesting that altered wood composition compromises xylem water transport and drought resistance.

### *PtoDUF1635* regulates vessel development and is negatively correlated with drought tolerance

To investigate the function of *PtoDUF1635*, we generated *PtoDUF1635* overexpression (*PtoDUF1635‐OE*) lines (Figure 6o; Figure S8a). *PtoDUF1635‐OE* lines showed no basal stem diameter differences under normal conditions but exhibited altered xylem anatomy: vessel lumen area increased by 23.4% and vessel density decreased by 13.8% compared to WT, resembling *PtobZIP18‐OE* phenotypes (Figure S8p–s). Wood composition (cellulose, hemicellulose, lignin) remained unchanged (Figure S8b–d), suggesting *PtoDUF1635* specifically regulates vessel development rather than cell wall biosynthesis.

To explore the roles of *PtoDUF1635* in drought stress, short‐term drought treatment was conducted to evaluate the response of *PtoDUF1635* plants to water deficit. Free proline, H_2_O_2_ and MDA contents in *PtoDUF1635‐OE* lines were similar to those of the *PtobZIP18‐OE* lines (Figure S8e–h), indicating that *PtoDUF1635* reduced xylem drought resistance.

Under long‐term drought, *PtoDUF1635‐OE* lines exhibited reduced basal stem diameters and increased PLD values compared to WT (Figure 6o,p; Figure S8i). Furthermore, cross‐sectional observations revealed that under drought stress, the mean vessel lumen area of the *PtoDUF1635‐OE* plants was 15.7% higher than that of the WT, while the mean vessel density was 18.2% lower (Figure 6q–s). Additionally, the water transport capacity, water potential and stem RWC of the *PtoDUF1635‐OE* lines were lower than those of the WT plants (Figure S8j–l). These findings imply that *PtoDUF1635* promotes the formation of vessels at lower density with larger lumen size, leading to lower water transport capacity and drought tolerance.

### *PtobZIP18* haplotypes are stable inheritances in hybrid populations

To further evaluate the breeding potential of *PtobZIP18* haplotypes, we conducted hybridization experiments involving 16 hybrid combinations (four parent combinations carrying the homozygous *PtobZIP18* ^*hap1*^ allele, 4 parent combinations carrying the homozygous *PtobZIP18* ^*hap2*^ allele, and eight parent combinations carrying the heterozygous allele were used, Data S8), resulting in 30 F1 hybrid populations. To assess the impact of environmental conditions on the growth of plants with different *PtobZIP18* haplotypes, we planted 30 F1 hybrid populations in low latitudinal locations (Liaocheng, Shandong province) and high latitudinal locations (Datong, Shanxi province), respectively. In Liaocheng, which has ample rainfall, the diameter at breast height (DBH)displayed a 7.1% increase in plants carrying the *PtobZIP18* ^*hap1*^ in a homozygous allele, demonstrated by DBH, compared to those carrying the *PtobZIP18* ^*hap2*^ in a homozygous allele, indicating that the *PtobZIP18* ^*hap1*^ has greater stem growth capacity (Figure S9a). Conversely, in Datong, where rainfall is limited, plants homozygous for *PtobZIP18* ^*hap1*^ exhibited a 16.5% decrease in DBH compared to those homozygous for *PtobZIP18* ^hap2^, suggesting that variation in precipitation contributes to growth differences between the two haplotypes (Figure S9a). Compared to the plants grown in Liaocheng, both haplotypes planted in Datong exhibited stem diameter loss, with *PtobZIP18* ^*hap2*^ showing a greater PLD of breast height (Figure S9b). These results imply that the *PtobZIP18* ^*hap1*^ would confer an advantage in production‐related applications, while the *PtobZIP18* ^*hap2*^ enhances drought resistance.

## Discussion

**Figure 7:**
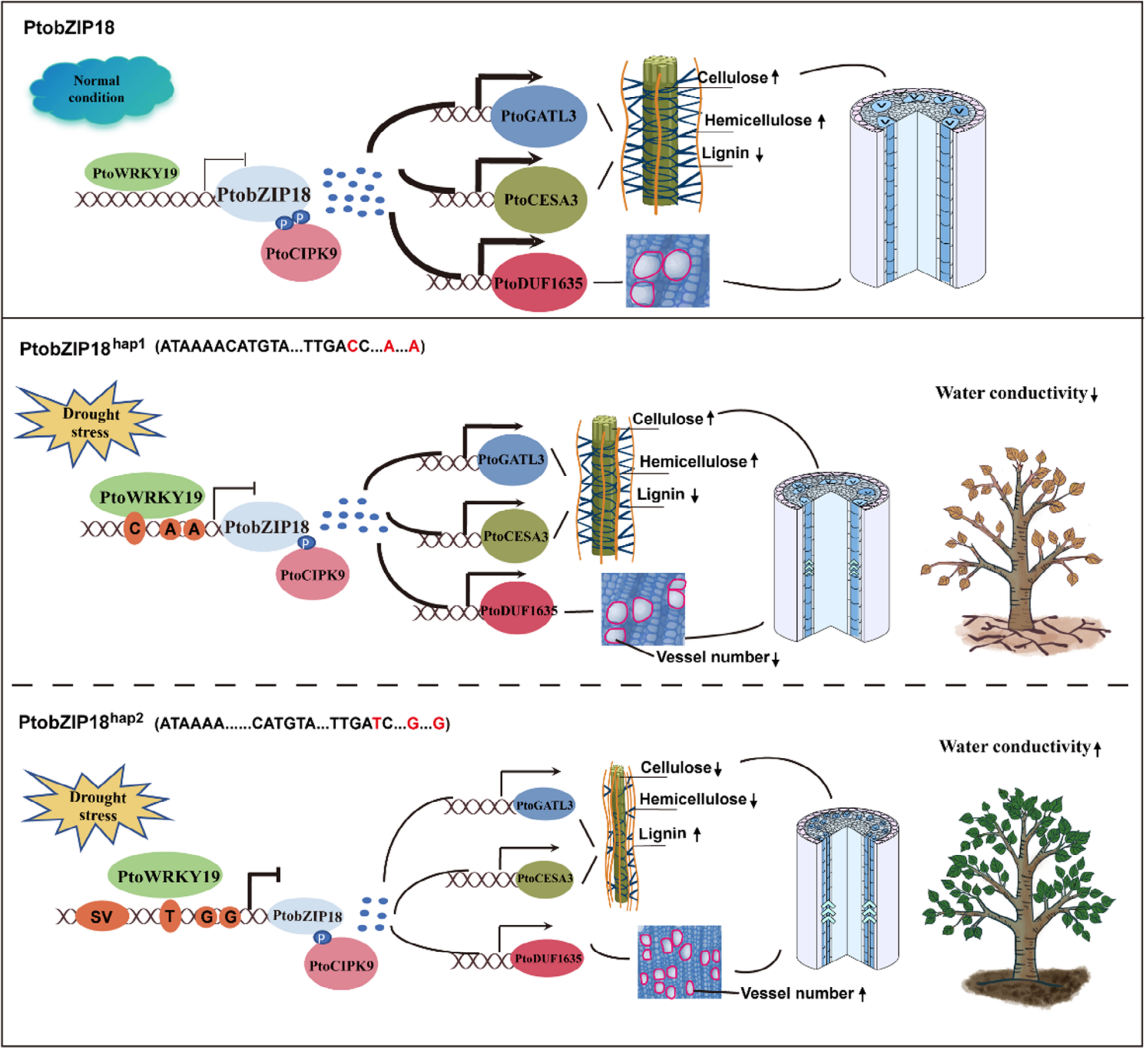
Proposed molecular model demonstrating that PtobZIP18 and its natural variants regulate the adaptive development of poplar under drought stress. Under normal conditions, *PtoWRKY19* exerts a negative regulation on the *PtobZIP18* gene, while the *PtobZIP18* gene positively regulates *PtoGATL3*, *PtoCESA3* and *PtoDUF1635*. During periods of drought stress, the drought response signals from *PtoWRKY19* suppress the expression levels of *PtobZIP18*; concurrently, drought signal activation of PtoCIPK9 phosphorylation ability decreases, which inhibits the PtobZIP18 protein stability and reduces its abundance. Furthermore, the two haplotypes of the *PtobZIP18* promoter exhibit distinct response patterns under drought stress. Mutations in the SV and SNP regions of the *PtobZIP18* ^*hap2*^ promoter, compared to the *PtobZIP18* ^*hap1*^ carrier, further reduce *PtobZIP18* ^*hap2*^ transcriptional activity, leading to lower expression of three downstream genes. This results in reduced cellulose and hemicellulose content, increased lignin content, higher vessel density, smaller vessel lumen area and enhanced water transport efficiency, ultimately improving drought resistance.

The xylem fulfils dual physiological roles, providing structural support for stem growth while facilitating long‐distance water transport through its specialized vessel network. Elucidating how key genes mediate the balance between wood development and the drought stress response to xylem adaptation is essential. We identified a module comprising PtoCIPK9/*PtoWRKY19*‐*PtobZIP18‐PtoGATL3/PtoCESA3/PtoDUF1635*, which revealed the role of *PtobZIP18* in regulating the adaptive development of secondary xylem at both the transcriptional and protein levels. Moreover, *PtoWRKY19* exhibited differential binding affinities to the two *PtobZIP18* promoter haplotypes, leading to allele‐specific regulation of *PtobZIP18* expression. This regulatory mechanism enables *PtobZIP18* to balance stem growth and drought resistance (Figure 7), providing insights into the evolutionary adaptation of trees to extreme drought.

### PtobZIP18‐*PtoGATL3/PtoCESA3/PtoDUF1635* inhibits water transport by regulating xylem development

As a homologue of *AtbZIP18*, *PtobZIP18* exhibits functional conservation within the bZIP transcription factor family—known regulators of vascular development and drought responses (Kong *et al*., 2023; Li *et al*., 2019; Yue *et al*., 2023). In this study, we demonstrated that *PtobZIP18* directly activates gene expression, serving as a key xylem balance regulator under drought stress (Figure 1). Our findings reveal its dual role: under drought stress, PtobZIP18 acts as a xylem‐specific rheostat, balancing growth and survival by suppressing drought‐responsive genes. This adaptive strategy carries ecological trade‐offs. While *PtobZIP18* overexpression enhances stem diameter under well‐watered conditions through xylem expansion, it concurrently increases embolism risk due to reduced vessel density and structural instability (Cai and Tyree, 2010; Hacke *et al*., 2006). Such hydraulic efficiency–safety conflicts highlight the evolutionary constraints shaping drought adaptation strategies in woody perennials.

This study showed that the transcription factor *PtobZIP18* activates *PtoGATL3*, *PtoCESA3*, and *PtoDUF1635* (Figure 4). *AtGATL3* encodes a glycosyltransferase family protein that plays a crucial role in modulating xylose and galactose (Kong *et al*., 2011), which are essential components of plant cell walls that contribute to their structural integrity and functionality (Lamport *et al*., 2011). Furthermore, genes related to xyloglucan metabolism play key roles in plant growth and stress resistance by altering the composition and structure of cell walls (Zhang *et al*., 2023). *AtCESA3* is a homologue of *PtoCESA3* in *Arabidopsis* that plays a pivotal role in regulating cellulose biosynthesis. Overexpression of *AtCESA3* results in higher cellulose content and thicker secondary walls (Burn *et al*., 2002; Zhang *et al*., 2022). However, excessive thickening of secondary cell walls reduces their flexibility, negatively impacting water absorption and transport, which explains the lower water conductivity observed in the *PtoCESA3* overexpression lines. Both *PtoGATL3* and *PtoCESA3* are critical in regulating the xylem composition, significantly influencing xylem development and secondary cell wall structure (Figure 6). These genetic factors modify not only xylem architecture but also water transport efficiency, highlighting their essential roles in plants' physiological adaptation.

As a member of the functionally uncharacterized DUF1635 family, PtoDUF1635's role in wood formation is inferred through its interaction networks. Previous studies position it within the poplar wood formation regulatory network (Petzold *et al*., 2018). In the study, we showed that *PtoDUF1635* plays a crucial role in regulating water transport capacity by modulating the structure of the vascular system under drought stress. However, to date, we have established only the interaction between *PtoDUF1635* and genes associated with vascular development; the precise molecular mechanisms underlying its function remain to be fully elucidated. As a previously underexplored gene, further investigation of *PtoDUF1635* will clarify its role in vascular system development and the regulation of water transport under drought stress, offering potential molecular targets for improving plant drought tolerance and optimizing water use efficiency.

### PtobZIP18 regulates adaptive xylem development through co‐regulation of protein phosphorylation and transcriptional activity

Among physiological and developmental processes in plants, transcriptional regulation and protein levels are crucial for maintaining cellular function and adaptability (Baillo *et al*., 2019; Pino *et al*., 2008; Tolosa and Zhang, 2020). Transcriptional regulation affects gene expression by enabling transcription factors to bind to specific DNA sequences, thereby initiating or repressing the transcription of target genes (Yin *et al*., 2023). Simultaneously, post‐translational protein modifications fine‐tune these biological processes by altering their activity, stability, and interactions with other molecules (Conibear, 2020; Lee *et al*., 2023). This intricate relationship ensures that plant cells respond rapidly and effectively to both environmental changes and internal signals. By coordinating these two types of regulation, plants optimize growth and development while adapting to varying conditions (Ichimaru *et al*., 2022).

Post‐translational modifications play a pivotal role in the physiological functions of *bZIP* transcription factors in plants. For example, SnRKs phosphorylate members of the bZIP family, such as ABFs, enhancing their transcriptional activity or protein stability in response to abiotic stress (Furihata *et al*., 2006; Kobayashi *et al*., 2005; Sirichandra *et al*., 2010). CIPK1, an important member of the CIPK family, interacts with the calcium sensors CBL1 and CBL9, facilitating the phosphorylation of bZIP transcription factors (Zhang *et al*., 2023). Our findings demonstrate that PtoCIPK9 phosphorylates the PtobZIP18 protein. Previous research in *Arabidopsis thaliana* showed that loss of CIPK9 function resulted in ABA‐hypersensitive stomatal closure and significantly enhanced drought tolerance (Song *et al*., 2018). Consistent with those findings, our study supports a model in which the protein kinase PtoCIPK9 acts as a drought sensor. By phosphorylating PtobZIP18, PtoCIPK9 destabilizes the protein, promoting its degradation and potentially modulating drought response mechanisms (Figure 5). Additionally, our study revealed other regulatory mechanisms that influence *PtobZIP18* activation at the transcriptional level. Under drought stress, *PtoWRKY19*, a homologue of *PtrWRKY19*, responded directly to drought signals, enhancing transcriptional regulation and inhibiting the promoter activity of *PtobZIP18*, thereby modulating the entire xylem development and drought response module in *P. tomentosa* (Figure 3). Additionally, *PtrWRKY19* recruited *PtrMYB074*, forming a dimer that activated *PtrbHLH18*, overexpression of which delays plant growth, increases stem‐to‐vessel ratios, and confers a strong drought‐tolerant phenotype (Liu *et al*., 2022). Similarly, the *PtoWRKY19‐OE* lines had significant drought tolerance, further emphasizing the critical role that this transcription factor plays in enhancing plant resilience under water‐limited conditions (Figure 3). The intricacy of this network of interactions highlights the complexity of the regulatory pathways that enable *P. tomentosa* to adapt and thrive in challenging environments.

Natural variation in promoter base mutations can significantly influence gene expression and organismal phenotypes, including traits such as cold tolerance and drought resistance. These variations regulate gene expression by altering transcription factor binding sites or enhancing the activity of specific promoter elements (Jiang *et al*., 2022; Sun *et al*., 2011). Moreover, increasing evidence implies that large SVs, such as presence/absence variants and copy‐number variants, play crucial roles in plants' adaptive evolution and functional diversity (Jayakodi *et al*., 2024; Qin *et al*., 2021; Wang *et al*., 2018). In this study, we found that sequence variations at SNP‐231, SNP‐260, SNP‐270 and SNP‐288 (one SV) in the *PtobZIP18* promoter determined the binding affinity of *PtoWRKY19*, leading to diverse *PtobZIP18* expression levels and drought‐tolerance variation in *P. tomentosa*. Under adequate‐moisture conditions, transcriptional inhibition of the interaction between *PtoWRKY19* and the promoter of the *PtobZIP18* ^*hap1*^ allele was weak, promoting plant growth and development. Conversely, in water‐limited environments, *PtoWRKY19* bound preferentially to the *PtobZIP18* ^*hap2*^ allele promoter, resulting in stronger transcriptional suppression. This suppressed *PtobZIP18* expression resulted in xylem structure adaptation and enhanced resilience under prolonged drought conditions. These adaptive responses enable plants to maintain physiological balance and improve their survival in challenging environments.

### Superior haplotype in *PtobZIP18* improves drought tolerance by modifying xylem structure

Drought resistance is a polygenic trait shaped by genomic‐environmental interactions (Blum, 2011). While reverse genetics approaches have identified key drought‐responsive genes in poplar (Li *et al*., 2019; Niu *et al*., 2024; Zhang *et al*., 2023), these studies often focus on single‐gene functions, overlooking the systemic regulation of drought adaptation. Moreover, most research has centred on leaves and roots, neglecting the xylem—a critical tissue for water transport and drought tolerance. To address this gap, we employed a phenotype‐based index (PLD) to assess xylem‐specific drought responses. Through GWAS, we identified *PtobZIP18* as a key candidate gene regulating xylem adaptation under drought (Figure 1a). This finding highlights the importance of xylem plasticity in drought resilience and provides a new target for improving poplar drought tolerance. *PtobZIP18* expression and regulatory mechanisms were influenced by SNPs and SV variants (Figure 3). This study identified a 110‐bp indel (SV) and three SNP variants in the *PtobZIP18* promoter region that divided a natural population of 300 *P. tomentosa* accessions into two haplotype groups: *PtobZIP18* ^*hap1*^ and *PtobZIP18* ^*hap2*^ (Figure 2). These haplotypes exhibited significant phenotypic differences.

Diverse ecological environments and natural genetic variation have led to the emergence and functionality of distinct haplotypes. These haplotypes play crucial roles in enabling plants to adapt to specific environmental conditions, allowing them to respond effectively to various ecological challenges (Cao *et al*., 2020; Liu *et al*., 2022; Sánchez‐Bermejo *et al*., 2014; Tang *et al*., 2018). The natural distribution of *P. tomentosa* reflects its capacity to tolerate drought stress (Figure 2). The *PtobZIP18* ^*hap1*^ haplotype is found primarily in southern China, where the climate is characterized by mild temperatures and abundant rainfall (Figure S3c). These favourable conditions support the rapid growth of *P. tomentosa* (Xiao *et al*., 2023). The high humidity and warmth increased trunk thickness, radial width of the secondary xylem and vessel diameters, which enhance water uptake and contribute to vigorous growth (Schuldt *et al*., 2010; Zhang *et al*., 2022). However, under drought stress, these larger vessels' structural instability increases their susceptibility to collapse, impairing water transport and leading to reduced growth, a decline in PLD and higher plant mortality (Figure 2). The *PtobZIP18* ^*hap2*^ haplotype is predominantly found in water‐scarce regions, such as the northeastern and northwestern climate zones of China. Populations in these areas are exposed to drought stress more frequently than are those in well‐hydrated environments, such as in the southern climate zone. As *P. tomentosa* grows and develops, it can adapt to challenging water conditions by acquiring drought resistance. This adaptation involves moderated inhibition of secondary xylem development while simultaneously stimulating the formation of the conduit structures that are essential for water transport (Keret *et al*., 2024). Stable and efficient water transport systems need to be established to meet the hydration needs of trees during normal growth. In the event of prolonged exposure to drought conditions, resilient structures develop that enhance stability, enabling the trees to withstand environmental pressures better (Chenlemuge *et al*., 2015). Under drought stress, the growth stability of the populations in these regions is enhanced, as characterized by lower PLD values and reduced mortality rates compared to those in the southern climate zone. This highlights the delicate balance in challenging environments between beneficial adaptations and inherent vulnerabilities. The drought tolerance conferred by the *PtobZIP18* ^*hap2*^ is likely an adaptive mechanism shaped through prolonged functional differentiation and natural selection.

Haplotype‐based hybrid populations provide a powerful approach to dissect genotype–phenotype relationships, offering insights into inheritance mechanisms and functional differentiation of alleles (Gou *et al*., 2024; Gu *et al*., 2023). Our results demonstrate that *PtobZIP18* ^*hap1*^ and *PtobZIP18* ^*hap2*^ exhibit distinct advantages under contrasting environments: *PtobZIP18* ^*hap1*^ enhances growth under water‐sufficient conditions, while *PtobZIP18* ^*hap2*^ improves drought resistance, reducing PLD by 16.2% compared to hap1 (Figure S9). This demonstrates that *PtobZIP18* ^*hap2*^ enhances drought resistance, likely through mechanisms that prioritize water conservation and stability in secondary xylem structure. These findings highlight the potential of *PtobZIP18* as a key genetic target for improving poplar resilience and productivity. The distinct roles of *PtobZIP18* ^*hap1*^ and *PtobZIP18* ^*hap2*^ underline the importance of considering environmental context when selecting genetic traits for breeding programmes. The ability to select for both high productivity and enhanced drought resistance through haplotype‐based breeding could greatly enhance the adaptability and sustainability of poplar cultivation in the face of climate change.

## Materials and methods

### Sample collection and plant materials

A natural population of 303 *P. tomentosa* accessions from southern, northwestern, and northeastern China (Du *et al*., 2012; Du *et al*., 2015) was established in Shandong (2009). From these, 300 grafted clones (three replicates/accession) were exposed to drought for 4 months: soil moisture reduced from 75%–80% to 20%–25% for 1 month before phenotyping. Detailed methods are described in Method S1.

### Phenotypic data of the *P. tomentosa* association population

We measured the basal stem traits under watering and drought stress as *D*_max_ and *D*_min_, respectively. The PLD of the basal stem was calculated as: PLD = 100 * (*D*_max_–*D*_min_)/*D*_max_. We also measured the contents of cellulose, hemicellulose, and lignin in wood components, as well as vessel density and diameter under well‐watered conditions. Detailed methods are available in Method S2.

### Genomic resequencing and variant calling

Genome re‐sequencing of the 300 selected accessions was conducted using the Illumina GA2 sequencing platform, achieving genomic re‐sequencing and variant calling depth of >30× (raw data) according to the manufacturer. SNPs and InDels were annotated using SnpEff (Cingolani *et al*., 2012).

### GWAS analysis

A total of 952 381 SNPs (MAF >5%, missing rate <20%) were analysed using a mixed linear model in EMMAX, with a genetic relatedness matrix to account for population structure. Genome‐wide significance was determined by Bonferroni correction (*P* < 1.05 × 10^−6^). Detailed methods are described in Method S3.

### Sequence alignment and phylogenetic analysis

Sequence alignment and phylogenetic analysis are described in Method S4.

### Gene cloning and constructs

We constructed full‐length CDSs of *PtobZIP18, PtoCESA3, PtoGATL3, PtoDUF1635*, and *PtoWRKY19* onto the pBI121 vector. The primers used for vector construction are provided in Table S1. The detailed carrier construction process is in Method S5.

### Plant materials and growth conditions

*Populus alba × P. glandulosa cv*. “84 K” was transformed using established protocols. Transgenic lines were acclimated in a mist chamber (30 days) and transferred to a greenhouse (3 months) for phenotypic analysis under randomized design. All plants were maintained at 25 °C, 60% RH, with 16‐h light/8‐h dark cycles.

### Drought treatment

Short‐term: Three‐month‐old seedlings underwent a 10‐day drought (SRWC reduced from 70% to <25%; controls ≥70%). Long‐term: 1‐month‐old seedlings acclimated for 50 days, then were exposed to a 40‐day drought (SRWC 20%–25%; controls ≥70%). Detailed methods are described in Method S6.

### Measurement of drought‐related indicators

The measurement of drought‐related indicators is described in Method S7.

### RNA‐seq analysis

RT‐PCR is described in Method S8.

### Evaluation of gene expression using RT‐qPCR

Total RNA was extracted from root, xylem, phloem, cambium, leaves and apex. RT‐qPCR was performed using SYBR Premix Ex Taq™ (TaKaRa) on the 7500 Fast Real‐Time PCR System. Gene expression was calculated using the 2^−∆∆Ct^ method with actin as the internal control. A detailed RT‐qPCR method is described in Method S9.

### GUS analysis

Promoters of *PtoWRKY19* were cloned into *pCAMBIA2300*. The *PtoWRKY19pro*:GUS lines were constructed using WT poplar. As described previously (He *et al*., 2018), GUS staining was performed on the 10th internode of the stem at 0 and 8 days of drought. GUS staining was carried out using a β‐glucuronidase Reporter Gene Staining Kit (Biorigin, Beijing, China) according to the manufacturer's instructions. The stained tissue was then captured with a microscope camera (Leica, Germany).

### Subcellular localization analysis

Constructs were transformed into Agrobacterium GV3101 (OD ~ 600 = 0.8) and infiltrated into tobacco leaves. After 72 h, GFP signals were visualized by confocal microscopy. Primers are listed in Table S1. Detailed methods are described in Method S10.

### Histochemical and histological analysis

Histochemical and histological analysis is described in Method S11.

### Luciferase complementation imaging assays (LCI)

Constructs were co‐transformed into tobacco leaves, and luciferase activity was measured using the Dual‐Luciferase Reporter Assay System (Vazyme). Primers are listed in Table S1. Detailed methods are in Method S12.

### Yeast one‐hybrid assay(Y1H)

Promoters of *PtobZIP18, PtoGATL3, PtoCESA3*, and *PtoDUF1635* were cloned into pAbAi and transformed into Y1H Gold yeast as baits. Positive interactions were confirmed by growth on SD/−Leu with AbA. Primers are listed in Table S1. Detailed methods are in Method S13.

### Electrophoretic mobility shift assay (EMSA)

*PtoWRKY19* and *PtobZIP18* CDSs were cloned into *pET‐32a* for His‐tagged protein expression in *E. coli* BL21 (DE3). EMSAs were performed using a Light Shift Chemiluminescent EMSA Kit (Beyotime). Probe sequences are presented in Table S1. Detailed methods are in Method S14.

### SPR‐based intermolecular binding assay

PtoWRKY19‐His was immobilized on CM5 chips, and SPR binding assays were performed with *PtobZIP18* ^*hap1*^ and *PtobZIP18* ^*hap2*^ oligonucleotides (0.54–20.95 μM) using a Biacore X100 platform. Primers are listed in Table S1. Detailed methods are in Method S15.

### Chip‐qPCR

Chromatin immunoprecipitation (ChIP) assays were conducted as described previously (Hu *et al*., 2022). The primers used for vector construction are provided in Table S1. Detailed methods are in Method S16.

### Yeast two‐hybrid assays (Y2H)

PtobZIP18 (bait) and PtoCIPK9 (prey) were cloned into *pGBKT7* and *pGADT7*, respectively, and co‐transformed into Y2H Gold yeast. Interactions were validated by growth on SD/−Trp/−Leu and SD/−Trp/−Leu/−His/−Ade media supplemented with X‐α‐gal. The primers used for vector construction are provided in Table S1. Detailed methods are in Method S17.

### Split luciferase (split‐LUC) complementation assay

PtobZIP18‐NLuc and PtoCIPK9‐CLuc fusion constructs were generated in *pCAMBIA1300*. Vectors were transiently expressed in *N. benthamiana* leaves via Agrobacterium GV3101 infiltration. Luciferase activity was imaged after 2 days (22 °C) using a molecular imaging system. Primers are listed in Table S1. Detailed methods are in Method S18.

### *In vitro* phosphorylation assay

Recombinant GST‐PtoCIPK9 and HIS‐PtobZIP18 were incubated in reaction buffer at 30 °C for 2 h. Phosphorylation signals were detected via autoradiography (Typhoon 9410). Primers are listed in Table S1. Detailed methods are in Method S19.

### LC–MS/MS assay

To identify the phosphorylation sites of PtobZIP18 by PtoCIPK9, an LC–MS/MS assay was conducted by Beijing BaitAIPAIke Biotechnology Co., Ltd. The detailed methods are described in Method S20.

### Statistical methods

Student's *t*‐test was performed using SPSS software (v.26.0) to determine significance, which was defined as *P < 0.05 and **P < 0.01. Immunoblot and EMSA results were quantified using ImageJ software.

### Accession numbers

All transcriptome expression data reported here have been submitted to the GSA under accession number (PRJCA036534).

## Conflict of interest

The authors declare no competing interests.

## Authors contributions

Q.D., L.L. and D.Z. designed the research. Z.J., R.H., M.Z., D.Z., M.Y. and J.D. performed experiments. Z.J. and P.L. collected and analysed the data; Z.J., P.L., L.L. and Q.D. wrote the manuscript; J.Z., W.Z., P.L., L.J., M.Q., L.D. and D.Z. provided valuable suggestions. Q.D. obtained funding and is responsible for this article. All authors read and approved the manuscript.

## Supporting information


**Figure S1** Identification and analysis of candidate genes.
**Figure S2** Morphology and physiology characteristics of WT, *PtobZIP18‐OE* and *PtobZIP18‐RNAi* transgenic poplars under long‐term and short‐term drought stress.
**Figure S3** Vessel phenotype and geographical distribution of different *PtobZIP18* haplotypes.
**Figure S4** PtoWRKY19 directly regulates the expression of *PtobZIP18*.
**Figure S5** PtobZIP18 activates the expression of *PtoGATL3, PtoCESA3* and *PtoDUF1635*.
**Figure S6** The Ser51 site of PtobZIP18 is phosphorylated by PtoCIPK9.
**Figure S7** Morphology and physiology characteristics of WT, *PtoGATL3*‐OE and *PtoCESA3‐OE* transgenic poplars under long‐term and short‐term drought stress.
**Figure S8** Morphology and physiology characteristics of WT, *PtoDUF1635*‐OE transgenic poplars under long‐term and short‐term drought stress.
**Figure S9** Natural variation in *PtobZIP18* promoter contributes to basal stem growth rate.
**Method S1** Sample collection and plant materials.
**Method S2** Phenotypic data of the *P. tomentosa* association population.
**Method S3** GWAS analysis.
**Method S4** Sequence alignment and phylogenetic analysis.
**Method S5** Gene cloning and constructs.
**Method S6** Drought treatment.
**Method S7** Measurement of drought‐related indicators.
**Method S8** RNA‐seq analysis.
**Method S9** Evaluation of gene expression using RT‐qPCR.
**Method S10** Subcellular localization analysis.
**Method S11** Histochemical and histological analysis.
**Method S12** Luciferase complementation imaging assays (LCI).
**Method S13** Yeast one‐hybrid assay (Y1H).
**Method S14** Electrophoretic mobility shift assay (EMSA).
**Method S15** SPR‐based intermolecular binding assay (SPR).
**Method S16** Chip‐qPCR.
**Method S17** Yeast two‐hybrid assays (Y2H).
**Method S18** Split luciferase (split‐LUC) complementation assay.
**Method S19** *In vitro* phosphorylation assay.
**Method S20** LC–MS/MS assay.
**Data S1** Variations in genomic region of PtobZIP18.
**Data S2** Gene information of the WRKY gene family different expressed genes between plants with Drought and Control.
**Data S3** Gene information of different expressed genes between transgenic plant and WT lines.
**Data S4** Gene information of different expressed genes between plants with Drought and Control.
**Data S5** PtobZIP18‐linked genes of the module contain.
**Data S6** Putative‐target gene information of PtobZIP18.
**Data S7** Gene information of yeast two‐hybrid screening using PtobZIP18 as bait protein.
**Data S8** Information on the PtobZIP18 gene and the 16 poplar parental hybrid combinations.
**Table S1** Oligonucleotide sequences of the primers used in this study.

## Data Availability

The raw data of genome re‐sequencing of 300 accessions of *Populus tomentosa* have been submitted to the Genome Sequence Archive (GSA) in the BIG Data Center under accession numbers CRA000903.
