# Supplementary material for: Natural variation in PtobZIP18 confers the trade‐off between stem growth and drought tolerance in Populus
Source: Plant Biotechnol J. 2025 Jul 13;23(10):4633–49. doi: 10.1111/pbi.70261 (PMC12483958; doi:10.1111/pbi.70261)
Supplement: Supplementary file 1 — Figure S1 Identification and analysis of candidate genes. Figure S2 Morphology and physiology characteristics of WT, PtobZIP18‐OE and PtobZIP18‐RNAi transgenic poplars under long‐term and short‐term drought stress. Figure S3 Vessel phenotype and geographical distribution of different PtobZIP18 haplotypes. Figure S4 PtoWRKY19 directly regulates the expression of PtobZIP18. Figure S5 PtobZIP18 activates the expression of PtoGATL3, PtoCESA3 and PtoDUF1635. Figure S6 The Ser51 site of PtobZIP18 is phosphorylated by PtoCIPK9. Figure S7 Morphology and physiology characteristics of WT, PtoGATL3‐OE and PtoCESA3‐OE transgenic poplars under long‐term and short‐term drought stress. Figure S8 Morphology and physiology characteristics of WT, PtoDUF1635‐OE transgenic poplars under long‐term and short‐term drought stress. Figure S9 Natural variation in PtobZIP18 promoter contributes to basal stem growth rate. Method S1 Sample collection and plant materials. Method S2 Phenotypic data of the P. tomentosa association population. Method S3 GWAS analysis. Method S4 Sequence alignment and phylogenetic analysis. Method S5 Gene cloning and constructs. Method S6 Drought treatment. Method S7 Measurement of drought‐related indicators. Method S8 RNA‐seq analysis. Method S9 Evaluation of gene expression using RT‐qPCR. Method S10 Subcellular localization analysis. Method S11 Histochemical and histological analysis. Method S12 Luciferase complementation imaging assays (LCI). Method S13 Yeast one‐hybrid assay (Y1H). Method S14 Electrophoretic mobility shift assay (EMSA). Method S15 SPR‐based intermolecular binding assay (SPR). Method S16 Chip‐qPCR. Method S17 Yeast two‐hybrid assays (Y2H). Method S18 Split luciferase (split‐LUC) complementation assay. Method S19 In vitro phosphorylation assay. Method S20 LC–MS/MS assay. Data S1 Variations in genomic region of PtobZIP18. Data S2 Gene information of the WRKY gene family different expressed genes between plants with Drought and Control. Data S3 Gene [file PBI-23-4633-s001.docx]

**Supplemental Figures**

**Figure S1** Identification and analysis of candidate genes.

**Figure S2** Morphology and physiology characteristics of WT, *PtobZIP18-OE*, and *PtobZIP18-RNAi* transgenic poplars under long-term and short-term drought stress.

**Figure S3** Vessel phenotype and geographical distribution of different *PtobZIP18* haplotypes.

**Figure S4** PtoWRKY19 directly regulates the expression of *PtobZIP18*

**Figure S5** PtobZIP18 activate the expression of *PtoGATL3,* *PtoCESA3* and *PtoDUF1635*.

**Figure S6** The Ser51 site of PtobZIP18 is phosphorylated by PtoCIPK9.

**Figure S7** Morphology and physiology characteristics of WT, *PtoGATL3*-OE and *PtoCESA3-OE* transgenic poplars under long-term and short-term drought stress.

**Figure S8** Morphology and physiology characteristics of WT, *PtoDUF1635*-OE transgenic poplars under long-term and short-term drought stress.

**Figure S9** Natural variation in *PtobZIP18* promoter contributes to basal stem growth rate.

**Supporting Methods**

**Method S1** Sample collection and plant materials

**Method S2** Phenotypic data of the *P. tomentosa* association population

**Method S3** GWAS analysis

**Method S4** Sequence alignment and phylogenetic analysis

**Method S5** Gene cloning and constructs

**Method S6** Drought treatment

**Method S7** Measurement of drought-related indicators

**Method S8** RNA-seq analysis

**Method S9** Evaluation of gene expression using RT-qPCR

**Method S10** Subcellular localization analysis

**Method S11** Histochemical and histological analysis

**Method S12** Luciferase complementation imaging assays (LCI)

**Method S13** Yeast one-hybrid assay(Y1H)

**Method S14** Electrophoretic mobility shift assay (EMSA)

**Method S15** SPR‑based intermolecular binding assay (SPR)

**Method S16** Chip-qPCR

**Method S17** Yeast two-hybrid assays (Y2H)

**Method S18** Split luciferase (split-LUC) complementation assay

**Method S19** LC-MS/MS assay

**Method S20** LC-MS/MS assay


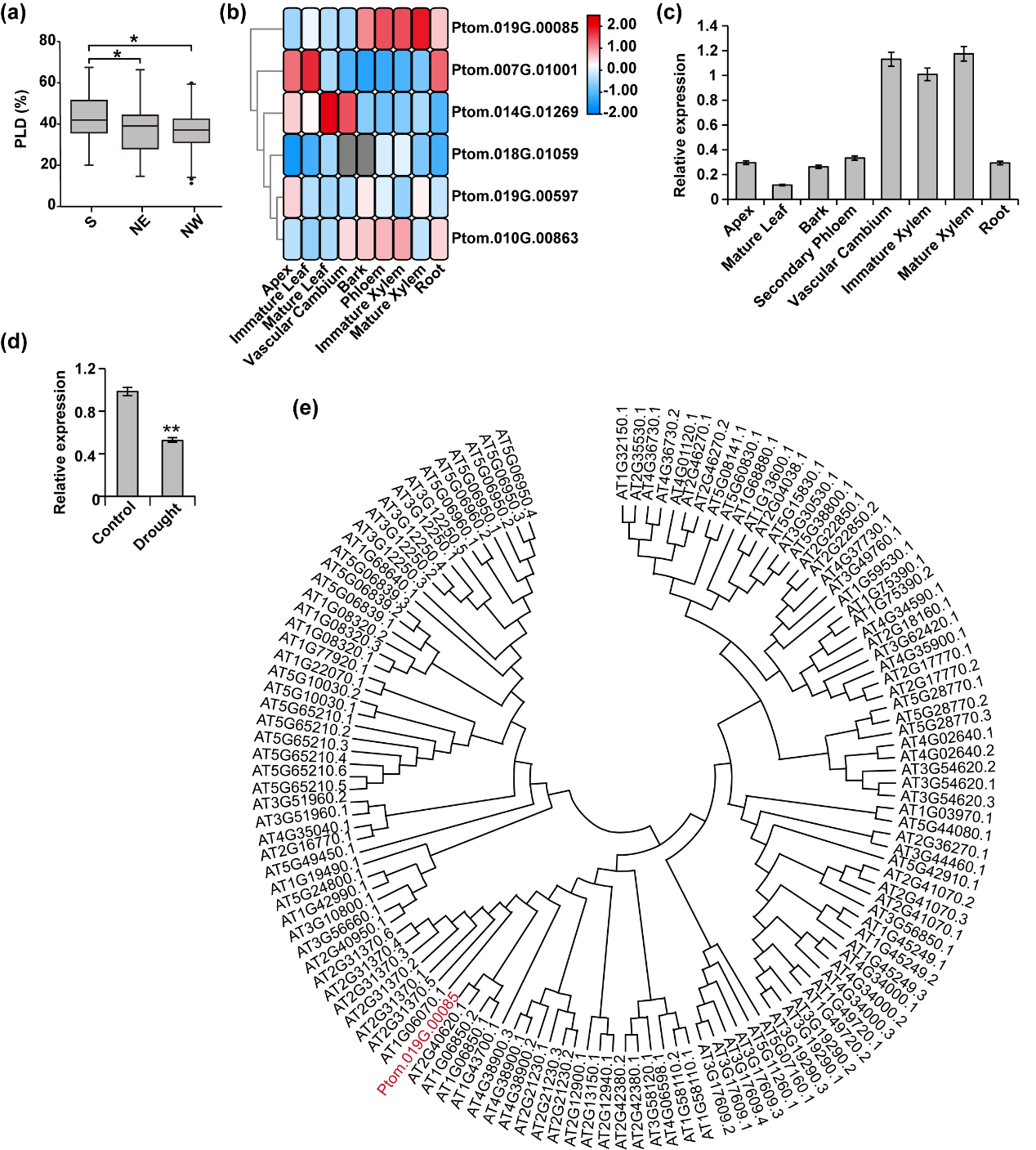


**Figure S1** Identification and analysis of candidate genes. (a) Comparison of PLD by three climatic zones. Values represent the mean ±SD of three biological replicates. Student’s t-test: *, *P* < 0.05; **, *P* < 0.01. (b) Expression patterns from nine tissues (bark, phloem, cambium, immature xylem, mature xylem, apex, immature leaf, mature leaf, and root) for annual poplar are shown in the heatmap. (c) Relative expression level of *PtobZIP18* in different tissues of 1-year-old poplar by RT-qPCR. (d) Relative expression level of *PtobZIP18* was determined by RT-qPCR in poplar with sufficient water and 40 days under drought stress, respectively. In (c) and (d) significant differences were determined using Student’s t-tests: *, *P* < 0.05; **, *P* < 0.01. Values are means ± SD. n = 3. (e) Phylogenetic consensus tree of PtobZIP18 proteins in *Populus tomentosa* (*Pto*) and *Arabidopsis thaliana* (*At*). The red texts indicate candidate genes in present study.


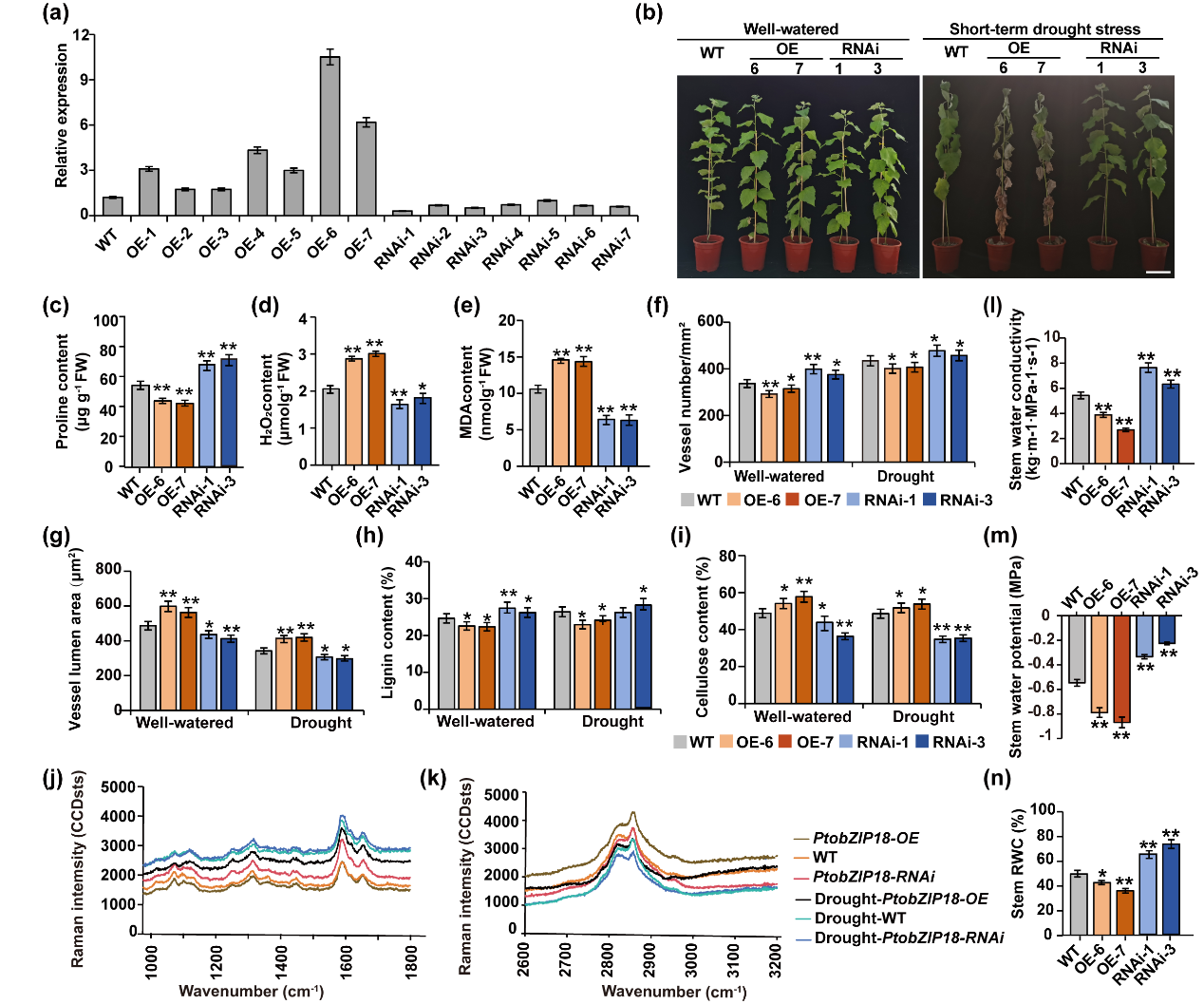


**Figure S2** Morphology and physiology characteristics of WT, *PtobZIP18-OE*, and *PtobZIP18-RNAi* transgenic poplars under long-term and short-term drought stress. (a) Relative *PtobZIP18* expression levels in the WT, individual *PtobZIP18-OE* transgenic lines (OE-1 ~ OE-7) and *PtobZIP18-RNAi* transgenic lines (RNAi-1 ~ RNAi-7), as determined by RT-qPCR. The values of WT were normalized to 1. Data represent the mean of three independent experiments ± SD. (b) Phenotypic comparison of WT, *PtobZIP18*-OE and *PtobZIP18-RNAi* plants grown in soil under drought conditions for 0 days and 10 days. Bars, 10 cm. Three individual poplars for each genotype were used and each showed similar results, with a representative picture showed. (c-e) Determination of proline content (c), hydrogen peroxide (H_2_O_2_) content (d), and malondialdehyde (MDA) content (e) in WT, *PtobZIP18*-OE and *PtobZIP18-RNAi* plants were measured after 0 and 10 days of drought stress. (f, g) Vessel number (f) and Vessel lumen area (g) from WT, *PtobZIP18-OE*, and *PtobZIP18-RNAi* poplar under well-watered condition and short-term drought stress. (h, i) Lignin content (h) and cellulose content (i) of plants under well-watered condition and long-term drought-stress condition. Data in (c-i) represent means ± SD of three independent experiments; statistical significance was assessed by Student’s t-test (**P* < 0.05, ***P* < 0.01). **(**j**)** Lignin Raman spectra analysis of vessel cell corners. 1600 cm^-1^ is the lignin Raman wavelength. **(**k**)** Cellulose Raman spectra analysis of vessel cell corners. 2900 cm^-1^ is the Raman wavelength of cellulose. (h-j). (l-n) Stem water conductivity (l), Stem water potential (m) and Stem RWC (n) under long-term drought-stress condition. Each mean and standard deviation were calculated from at least 3 plants. Asterisks show significant differences by Student’s t-test: *, *P* < 0.05; **, *P* < 0.01.


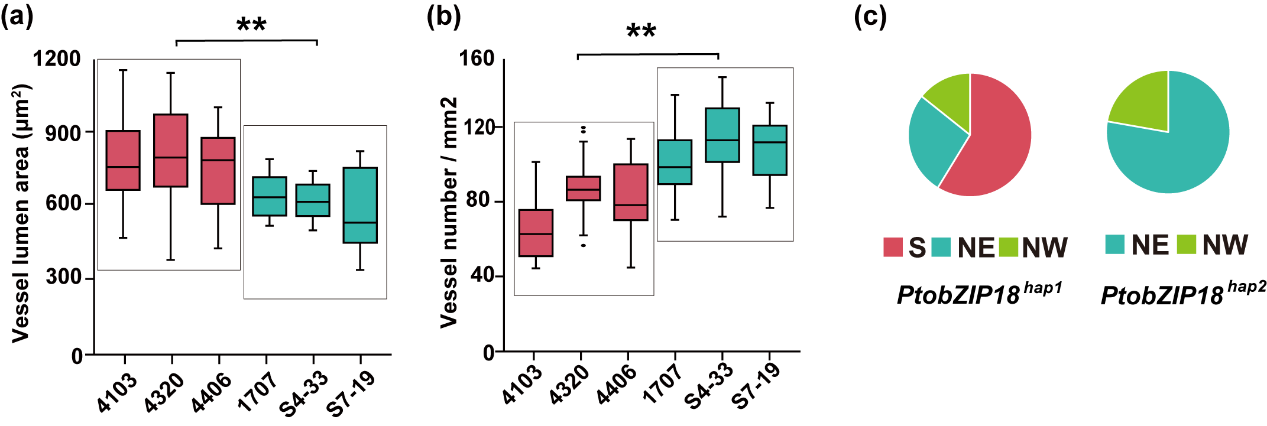


**Figure S3** Vessel phenotype and geographical distribution of different *PtobZIP18* haplotypes. (a, b) The six accessions were divided into *PtobZIP18^hap1^* and *PtobPZI18^hap2^* based the natural alleles of *PtobZIP18*. Vessel lumen area (a) and vessel density (b) of six accessions under well-watered condition. Values represent the mean ± SD of three biological replicates. Asterisks show significant differences by Student’s t-test: *, *P* < 0.05; **, *P* < 0.01. (c) Geographic distribution of *PtobZIP18* haplotypes in three different ecoregions. The pie charts are percentages of *PtobZIP18^hap1^* and *PtobPZI18^hap2^* in the three climate zones, respectively.


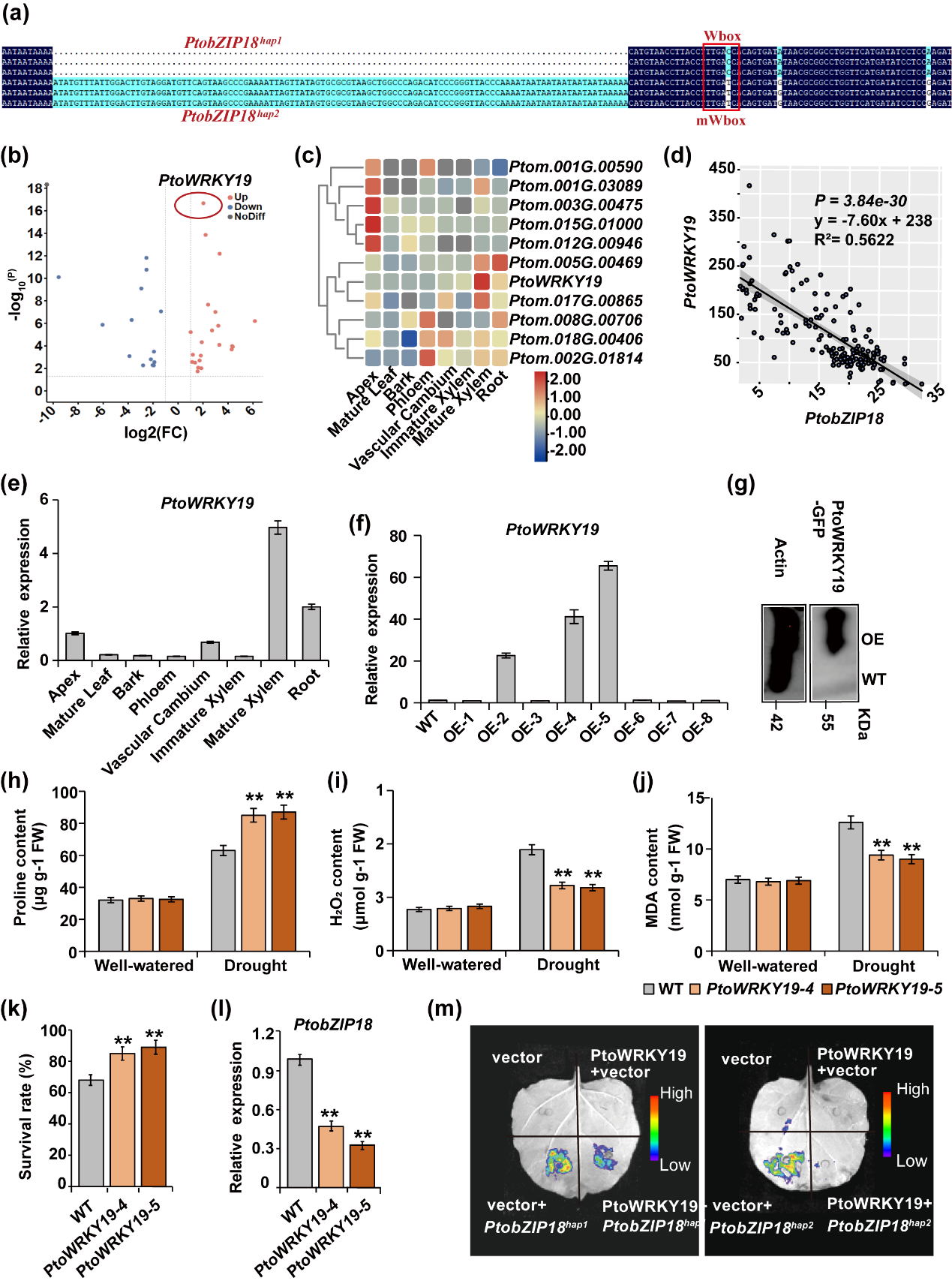


**Figure S4** PtoWRKY19 directly regulates the expression of *PtobZIP18*. (a) Promoter sequence comparison of *PtobZIP18^hap1^* and *PtobPZI18^hap2^*. Black boxes represent the same nucleic acid sequence. Green boxes represent different nucleic acid sequences. Red box represents W-box. (b) Volcano plots representing the fold-change of DEGs in the comparison groups of WRKY transcription factor in Drought versus Control. (*P* < 0.05, absolute fold change ≥ 2.0). (c) Heatmap showing the level of gene expression in each tissue. The color of heatmap (dark blue to red) indicates the correlation (low to high). (d) Correlation analysis of *PtobZIP18* and *PtoWRKY19* expression levels. (e) Relative expression level of *PtoWRKY19* in different tissues of 1-year-old poplar by RT-qPCR. (f) Expression of *PtoWRKY19* in transgenic Populus. The poplar actin was used as an internal standard for data normalization. Values are means ± SD. n=3. (g) *PtoWRKY19* protein abundance in WT and PtoWRKY19-GFP-overexpressing plants (*PtoWRKY19-5*). *PtoWRKY19* was detected with anti-GFP antibody. Actin served as a control. (h-j) Determination of proline content (h), hydrogen peroxide (H_2_O_2_) content (i), and malondialdehyde (MDA) content (j) in WT and *PtoWRKY19*-OE plants were measured after 0 and 10 days of drought stress. (k) Statistical analysis of survival rates after drought treatment and recovery (rehydrated for 2 d). (l) Expression of *PtobZIP18* in *PtoWRKY19*-OE *Populus*. The poplar actin was used as an internal standard for data normalization. Statistical analysis was performed with Student’s t-test (**P* < 0.05, ***P* < 0.01) in (h-l). Values are means ± SD. n = 3. (m) Relative luciferase activity according to a DLRA assay of *N. benthamiana* leaves. Luciferase activity assay showing that PtoWRKY19 binds to the promoter regions of *PtobZIP18^hap1^* and *PtobPZI18^hap2^*. The color-coded bar indicates the intensity of luciferase activity.


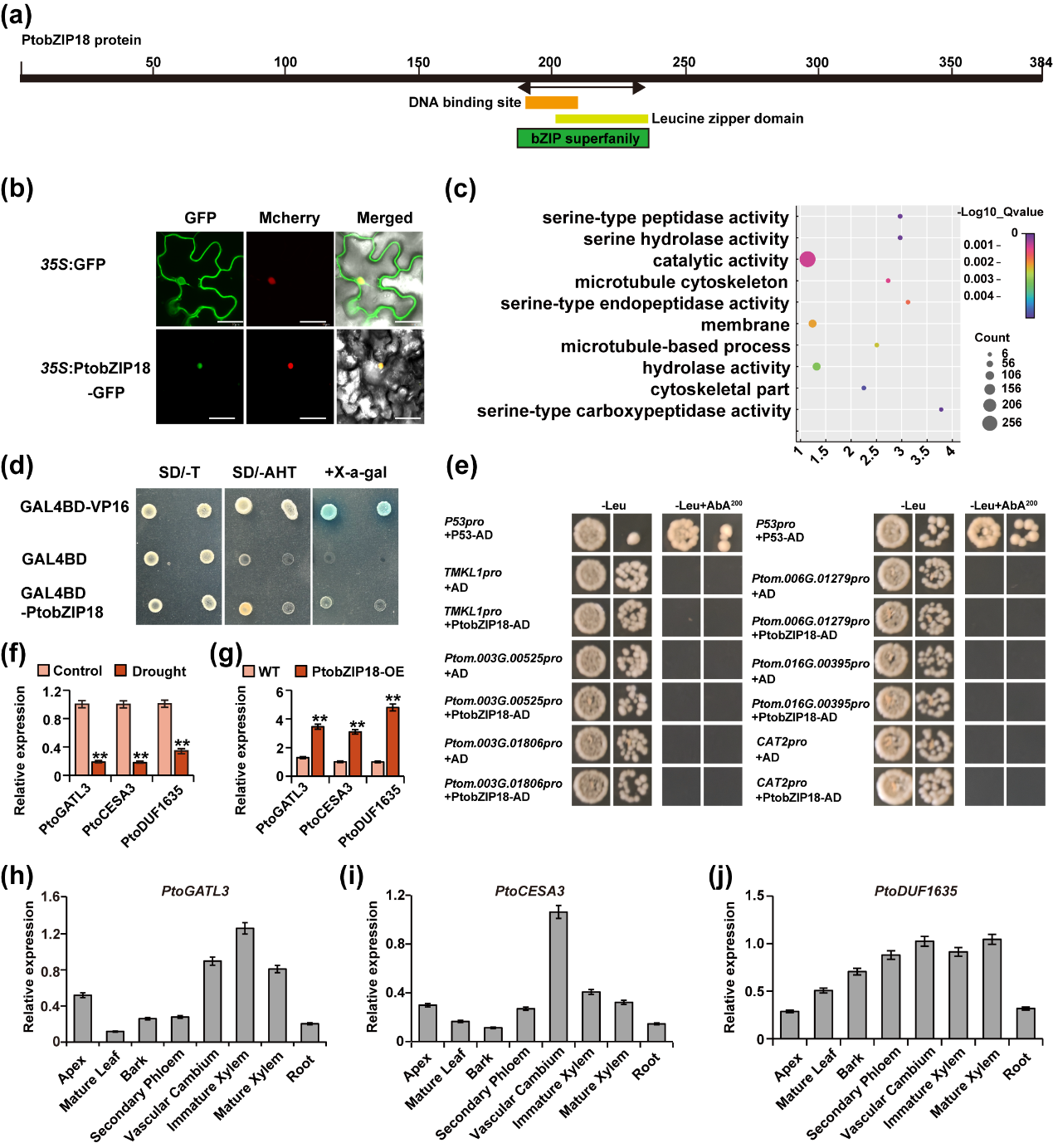


**Figure S5** PtobZIP18 activate the expression of *PtoGATL3,* *PtoCESA3* and *PtoDUF1635*. (a) Protein structure analysis of PtobZIP18. The DNA binding site and Leucine zipper domain are indicated in orange and light green, respectively. (b) Subcellular localization of *PtobZIP18*, *35S*: GFP and *35S*: PtobZIP18-GFP translational fusion constructs. mCherry served as a nucleus marker. Bar, 20 μm. (c) Gene Ontology (GO) categorization of common DEGs. Terms with large gene hit and low *P*-adjust were indicated. The circles represent the hit gene number and the colors represent the *P*-adjust. (d) Transcription activation activity of *PtobZIP18*. GAL4 DNA binding domain was fused with the full-length construct of *PtobZIP18* were expressed in the yeast strain Y2H Gold. Transformed yeast was grown in either SD/-Trp or SD/-Trp/-His/-Ade media. LacZ activity was observed in SD/-Trp medium containing X-α-Gal. pGBKT7 was used as a negative control. BD represents the GAL4 DNA binding domain. (e) Yeast one-hybrid assay showing that *PtobZIP18* binds to promoter of seven genes. AD-Rec-P53 and P53-promoter-AUR1-C were used as the positive controls, AD-Empty prey vector and AUR1-C under the control of one target gene promoter vector are used as the negative controls. Aureobasidin A (AbA), a yeast cell growth inhibitor, was used as a screening marker. (f) Expression of *PtoGATL3,* *PtoCESA3* and *PtoDUF1635* in *PtobZIP18*-OE plants. The poplar actin was used as an internal standard for data normalization. (g) Relative expression level of *PtoGATL3,* *PtoCESA3* and *PtoDUF1635* were determined by RT-qPCR in poplar with sufficient water and 40 days under drought stress, respectively. In (f-g), statistical analysis was performed with Student’s t-test (**P* < 0.05, ***P* < 0.01). Values are means ± SD. n = 3. (h-j) Relative expression level of *PtoGATL3* (h)*,* *PtoCESA3* (i) and *PtoDUF1635* (j) in different tissues of 1-year-old poplar by RT-qPCR.


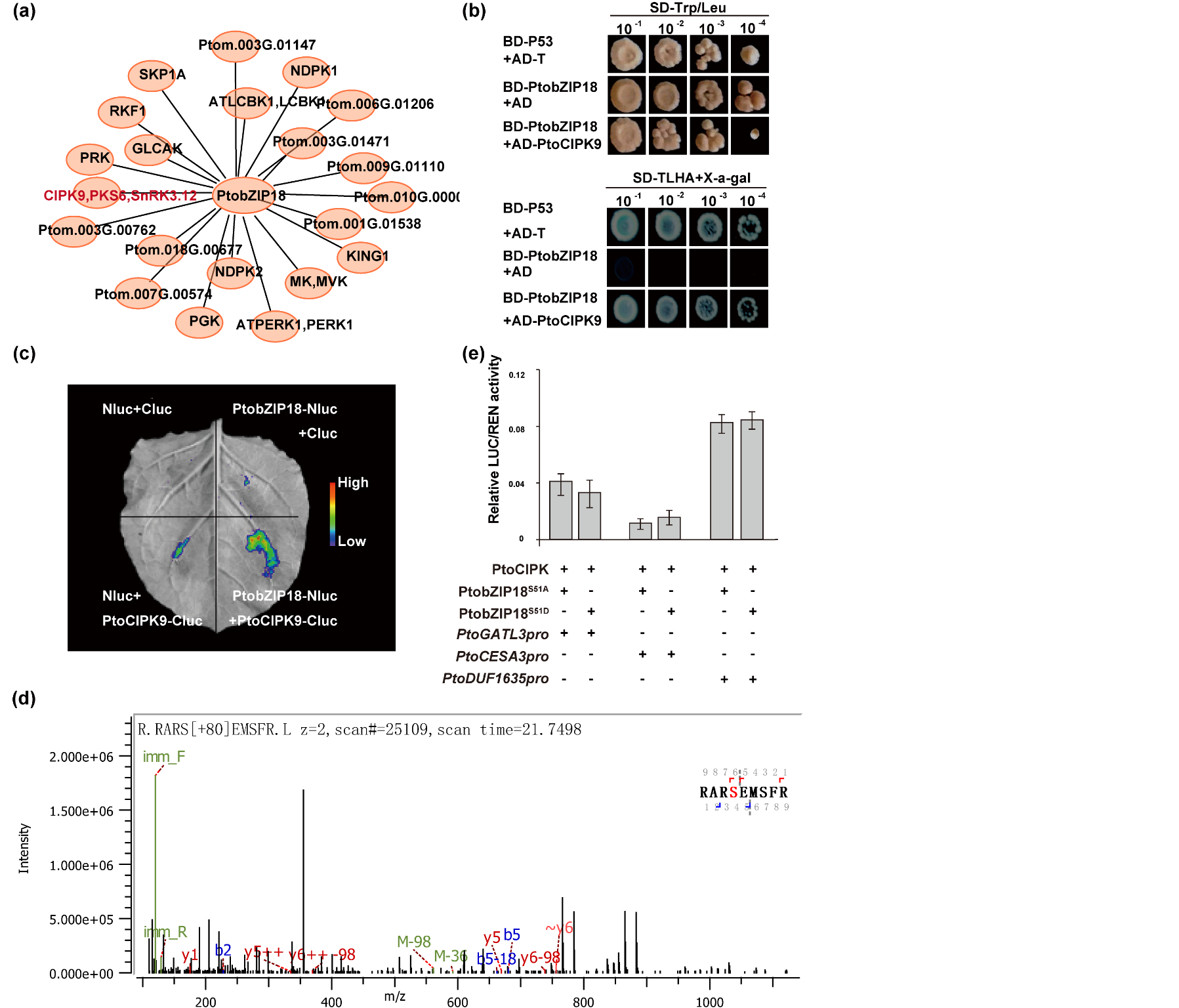
**Figure S6** The Ser51 site of PtobZIP18 is phosphorylated by PtoCIPK9. (a) The 21 kinases interacting with PtobZIP18 screened by yeast two-hybrid. (b) PtoCIPK9 interact with PtobZIP18 in yeast. Yeast cells were grown on synthetic defined (SD) medium lacking Leu and Trp (–Leu –Trp) or SD –Leu –Trp –His –Ade medium. (c) Split-LUC assay showing the interaction between PtobZIP18 and PtoCIPK9 in *N. benthamiana* leaf cells. (d) Liquid chromatography-tandem mass spectrometry (LC-MS/MS) showing the Ser51 of PtobZIP18 is phosphorylated by PtoCIPK9. Recombinant HIS-PtobZIP18 was incubated with GST-PtoCIPK9 at 30°C in protein kinase buffer containing 25 μmol/L ATP, and the reaction without ATP served as a negative control. (e) Relative luciferase activity according to a LCI assay of *N. benthamiana* leaves. Quantification was performed by normalizing firefly luciferase (LUC) activity to that of Renilla luciferase (REN), *35S*:REN was used as the internal control. Relative luciferase activities using PtobZIP18^S51A^-pGreenII62-SK as the effector were compared to the control effector (PtobZIP18^S51D^-pGreenII62-SK). PtoCIPK9 was overexpressed in tobacco cells driven by the 35S promoter. Error bars are ± SD. Statistical analysis was performed using the t-test (n = 3) and statistically significant differences are indicated by **P* < 0.05, ***P* < 0.01.


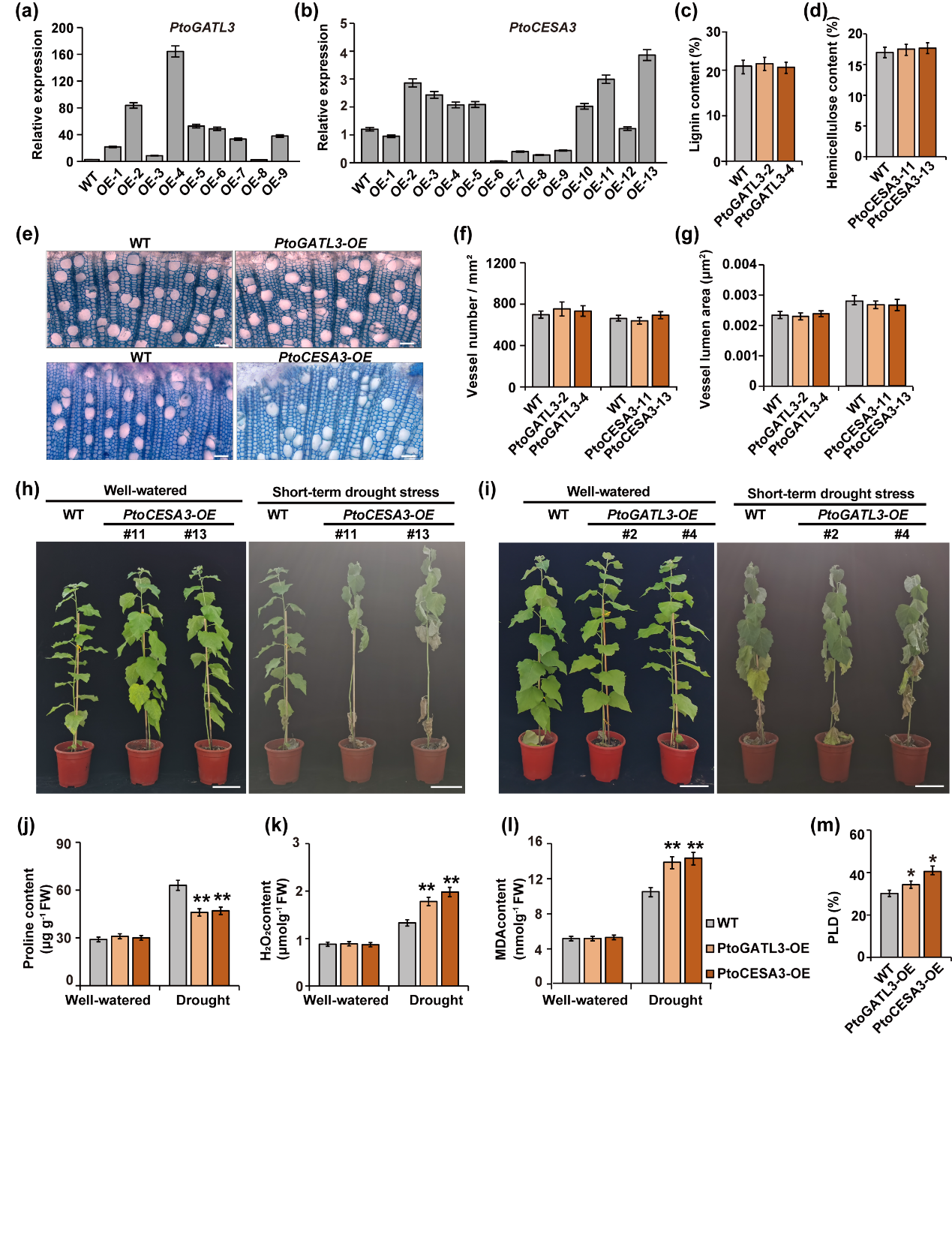
**Figure S7** Morphology and physiology characteristics of WT, *PtoGATL3*-OE and *PtoCESA3-OE* transgenic poplars under long-term and short-term drought stress. (a) Relative *PtoGATL3* expression levels in the WT, *PtoGATL3*-OE transgenic lines (OE-1～OE-9), as determined by RT-qPCR. (b) Relative *PtoCESA3* expression levels in the WT, *PtoGATL3*-OE transgenic lines (OE-1～OE-13), as determined by RT-qPCR. The values of WT were normalized to 1. Data represent the mean of three independent experiments ± SD. (c) Lignin content in WT and *PtoGATL3-OE* plants under well-watered condition. (d) Hemicellulose content in WT and *PtoCESA3-OE* plants under well-watered condition. (e) Vessel structure of mature xylem. Scale bar, 50 μm. Three independent experiments with three individuals each replicate is performed and each showed similar results, with a representative picture showed. (f, g) vessel lumen area (f), and vessel number (g) from 3-month-old *PtoGATL3*-OE and *PtoCESA3-OE* under well-watered condition. Values represent the mean ±SD of three biological replicates. Student’s t-test: *, *P* < 0.05; **, *P* < 0.01. (h, i) Phenotypic comparison of WT, *PtoGATL3*-OE (h) and *PtoCESA3-OE* (i) plants grown in soil under drought conditions for 0 days and 10 days. Bars, 10 cm. Three individual poplars for each genotype were used and each showed similar results, with a representative picture showed. (j-l) Determination of proline content (j), hydrogen peroxide (H_2_O_2_) content (k), and malondialdehyde (MDA) content (l) in WT, *PtoGATL3*-OE and *PtoCESA3-OE* plants were measured after 0 and 10 days of drought stress. (m) PLD trait in WT, *PtoGATL3*-OE and *PtoCESA3-OE* plants were measured after 40 days of drought stress. Three independent experiments were performed. Statistical analysis was performed with Student’s t-test (**P* < 0.05, ***P* < 0.01); data are provided as means ± SDs.


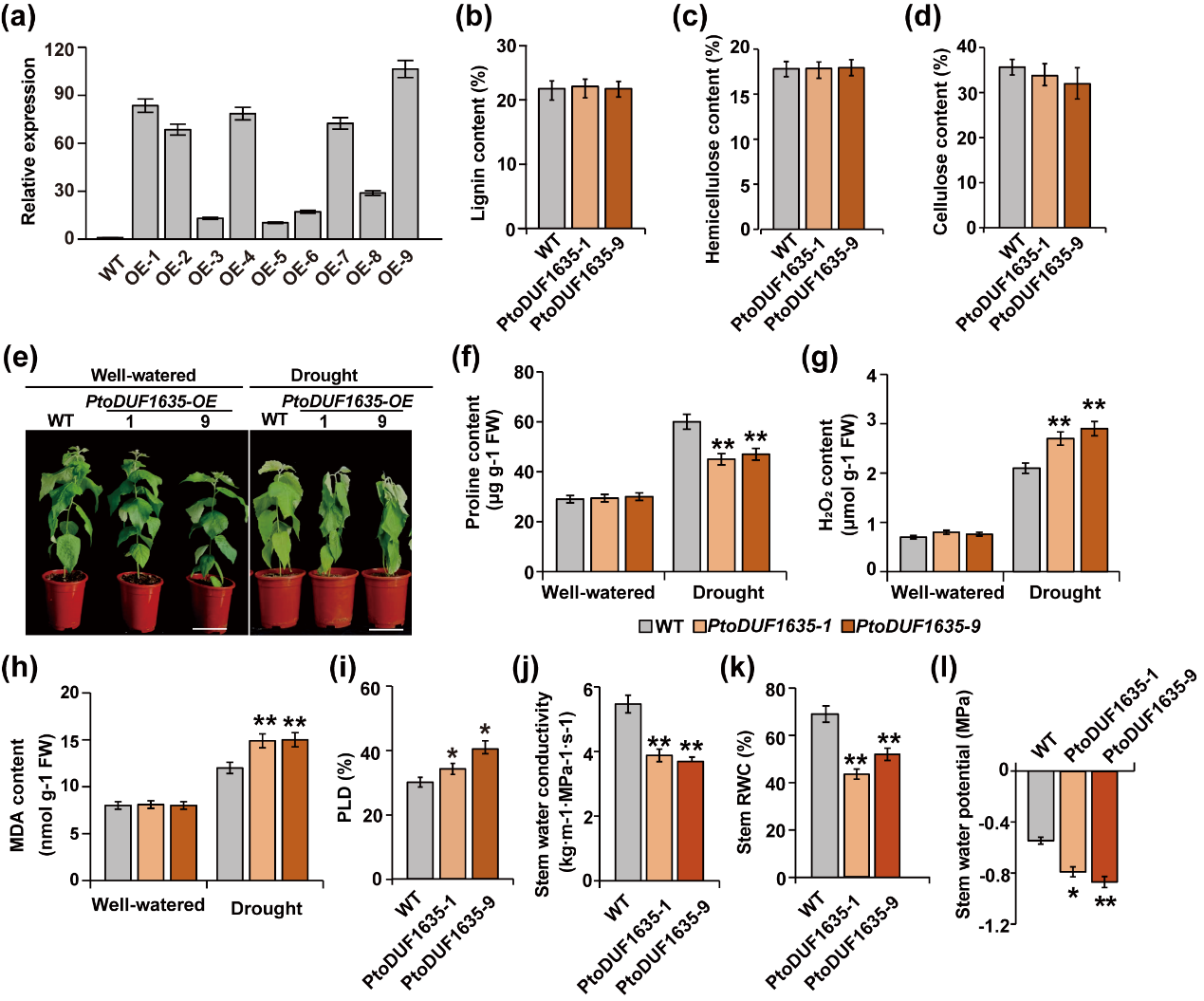


**Figure S8** Morphology and physiology characteristics of WT, *PtoDUF1635*-OE transgenic poplars under long-term and short-term drought stress. (a) Relative *PtoDUF1635* expression levels in the WT, *PtoDUF1635*-OE transgenic lines (OE-1～OE-9). (b-d) Lignin content (b), cellulose content (c), and hemicellulose content (d) in WT and *PtoDUF1635-OE* plants under well-watered condition. (e) Phenotypic comparison of WT, *PtoDUF1635-OE* plants grown in soil under drought conditions for 0 days and 10 days. Bars, 10 cm. Three individual poplars for each genotype were used and each showed similar results, with a representative picture showed. (f-h) Determination of proline content (f), hydrogen peroxide (H_2_O_2_) content (g), and malondialdehyde (MDA) content (h) in WT and *PtoDUF1635-OE* plants were measured after 0 and 10 days of drought stress. (i) PLD trait in WT and *PtoDUF1635-OE* plants were measured after 40 days of drought stress. (j-l) Stem water conductivity (j)**,** stem relative water content (k)**,** and Stem water conductivity (l) of plants under drought-stress condition. Three independent experiments were performed. Statistical analysis was performed with Student’s t-test (**P* < 0.05, ***P* < 0.01); data are provided as means ± SDs.


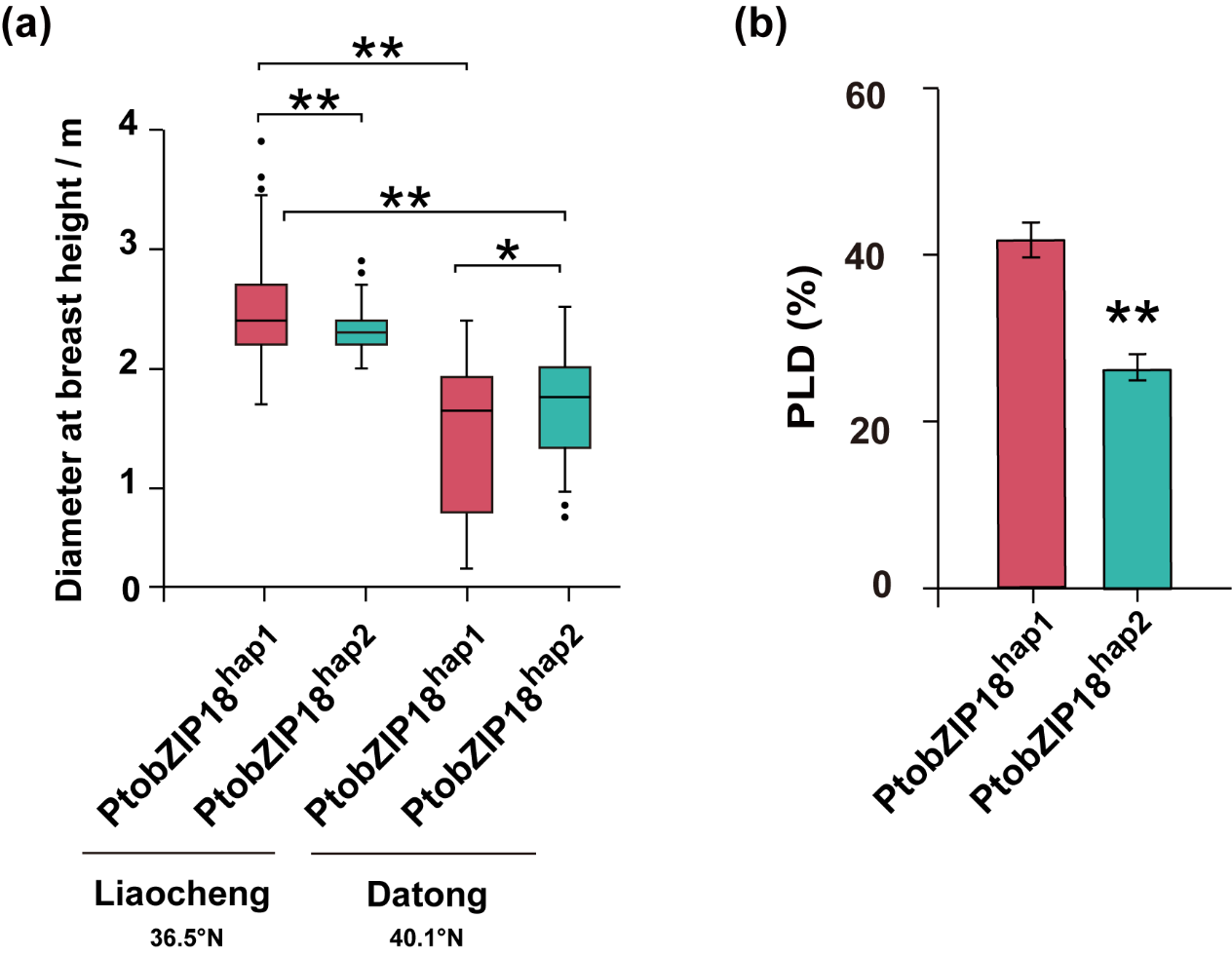


**Figure S9** Natural variation in *PtobZIP18* promoter contributes to basal stem growth rate. **(**a) The diameter at breast height (DBH) of plants grown in Liaocheng and Datong is categorized as follows: 0/0 and 1/1 denote two homozygous types; 0/1 indicates a heterozygous type. (b) PLD trait of *PtobZIP18^hap1^* and *PtobPZI18^hap2^* hybrid population. Values represent the mean ±SD of three biological replicates. Student’s t-test: *, *P* < 0.05; **, *P* < 0.01.

## Method S1 Sample collection and plant materials

The natural distribution of *P. tomentosa* (30–40° N, 105–125° E) was divided into southern, northwestern, and northeastern geographical regions based on the ISODATA fuzzy clustering of three comprehensive meteorological factors (Du *et al*., 2012). The 300 accessions used in this study were planted in Guan Xian County, Shandong Province, China (36°23'N, 115°47'E) in 2009. The accessions were categorized into three subpopulations based on geographic origin and genetic clustering: S (n = 84), NW (n = 108), and NE (n = 108) (Du *et al*., 2014). Over the previous 5 years, the annual average precipitation was higher in the S region than in the NW and NE regions (<http://www.cma.gov.cn/>)(Fang *et al*., 2023). An experimental population of 300 one-year-old accessions was asexually propagated via grafting, with three replicates per accession. Initially, soil moisture was maintained at 75–80%, but when the plants were 4 months old watering ceased and soil moisture fell to 20–25%. During this drought treatment, soil moisture was maintained at ≤ 25% for 1 month before trait evaluation.

## Method S2 Phenotypic data of the *P. tomentosa* association population

Stems from at least three independent individuals were collected, ground into fine powder in liquid nitrogen, passed through a 40-mesh screen (Yeh *et al*., 2005), and then analyzed. The AcBr method was used to quantify lignin content (Hatfield et al., 1999). The concentrations of monosaccharides (glucose and xylose) were analyzed by HPLC (Agilent 1260, USA) coupled with an Aminex HPX-87P column (300 × 7.8 mm, Bio-Rad Laboratories, USA) (Sluiter *et al*.**,** 2008). The transverse section of the stem was examined under a microscope, and the number of xylem vessels and their diameters were measured. Three biological replicates were performed.

## Method S3 GWAS analysis

For the GWAS analysis, 952,381 SNPs were used for association analysis, with a minor allele frequency of > 5% and a missing-data rate of < 20%. GWAS was performed using a mixed-model approach, implemented through the EMMAX software package. The matrix of pairwise genetic distances derived from the simple matching coefficients was used as the variance-covariance matrix for the random effects. The significance threshold was defined using Bonferroni correction, with the cutoff set as −log (1/total SNPs). The genome-wide significance level for branch number was determined to be 1.05 × 10^−6^.

## Method S4 Sequence alignment and phylogenetic analysis

The homologs of PtobZIP18 were downloaded from the NCBI nonredundant protein database (NR). The multiple sequence alignments were performed using MEGA v. 7, and the phylogenetic tree was constructed with MEGA7 using the neighbor-joining (NJ) method (Kumar *et al*., 2016). The reliability of the tree was estimated by bootstrapping using 2,000 replications.

## Method S5 Gene cloning and constructs

The full-length coding sequences (CDSs) of *PtobZIP18*, *PtoCESA3*, *PtoGATL3*, *PtoDUF1635*, and *PtoWRKY19* were amplified from *P. tomentosa* cDNA using 2 × Phanta Max Master Mix (Dye Plus) (P525-02; Vazyme, Nanjing, China) and specific primer pairs. These sequences were then cloned into the binary vector *pBI121*, driven by the 35S promoter. To generate RNAi constructs, a 200-bp fragment of PtobZIP18 was amplified from cDNA and inserted into the *pBI121* vector in an inverted repeat orientation. The stem-loop fragments were ligated into the modified *pBI121* vector, downstream of the 35S promoter, creating *PtobZIP18-RNAi* vectors.

## Method S6 Drought treatment

For the short-term drought treatment, 4-week-old tissue-culture seedlings were transplanted into pots and placed in a greenhouse at 23–25°C for 90 days, with a 16-hour light/8-hour dark photoperiod. Three-month-old poplars were subjected to drought stress, during which the soil relative water content (SRWC) was reduced from 70% to below 25% over a period of 10 days by withholding water. The SRWC of the control group was maintained above 70%.

Long-term drought stress experiments in soil were performed as previously described (Wang *et al*., 2016), with minor modifi-cations. Briefly, one-mouth-old tissue-culture seedlings were transplanted into pots and placed in a greenhouse at 23–25°C for 50 days, with a 16-hour light/8-hour dark photoperiod. Following this, the SRWC was maintained, and the drought trial was set at 20–25% for 40 days. The SRWC was maintained at ≥ 70% for controls, which has previously been shown to result in no water stress. To maintain SRWC, every pot was weighed daily, and lost water was supplemented as required. The SRWC was checked with a Soil moisture meter (Hengmei, Shandong, China), and checked by theoven-drying method.

## Method S7 Measurement of drought-related indicators

The contents of malondialdehyde, hydrogen peroxide and proline were measured with a detection kit (Comingbio, Suzhou, China), according to the manufacturer’s instructions. Stem RWC was measured by the oven-drying method. The stem water conductivity was measured using the XYLEM-Plus xylem water conductivity and embolism measurement system. Stem RWC was measured by the oven-drying method. Stem water potential was measured using Portable Plant Water Potential Pressure Chamber (1505DEXP, America), according to the manufacturer’s instructions (Li *et al*., 2019).

## Method S8 RNA-seq analysis

RNA-seq analysis was performed on total RNA extracted from the differentiating stem xylem of 3-month-old poplars. Stems from three different plants were pooled to create a single biological replicate. The transcriptome data were analyzed using established methods. RNA-seq data were generated using the Illumina HiSeq2000 platform at Novogene Ltd. (Beijing, China). To ensure data quality, we removed reads containing adapters, poly-N sequences, and low-quality reads from the raw data. The clean reads were then uniquely mapped to the *P. tomentosa* genome using TopHat 2.1.1 with default settings (Trapnell *et al*., 2009). Transcription levels were quantified using the reads per kilobase of transcript per million reads (RPKM) method. *P*-values were adjusted using the Benjamini and Hochberg method. Differentially expressed genes (DEGs) were identified using the DESeq2 package in R. A two-fold change [log2(fold change) ≥ 2] in gene expression levels, together with a q-value ≤ 0.05, was set as the threshold for identifying altered gene expression.

## Method S9 Evaluation of gene expression using RT-qPCR

To determine the tissue specificity of *PtobZIP18, PtoCESA3, PtoGATL3, PtoDUF1635*, and *PtoWRKY19* expression, a FastPure Plant Total RNA Isolation Kit (Vazyme, Beijing, China) was used to isolate total RNA from the root, mature xylem, immature xylem, vascular cambium, secondary phloem, old leaves (seventh to ninth leaves), and apex. All RNA was reverse transcribed into cDNA using the Reverse Transcription System (Promega Corporation, Madison, WI, USA). RT-qPCR was then performed on the 7500 Fast Real-Time PCR System using SYBR Premix Ex Taq™ Kit (TaKaRa, Dalian, China) according to the manufacturers’ instructions. The primers used for RT-qPCR are summarized in Table S1. The relative gene expression level was calculated using the 2^−∆∆Ct^ method. The actin gene was used as an internal control. All reactions were performed with at least four technical replicates, and the experiment was repeated three times. For gene-expression detection in developing xylem, samples were collected by scraping the surface of the debarked trunk.

## Method S10 Subcellular localization analysis

The full-length CDs of *PtobZIP18* was cloned into the *pBI121* vector to generate the recombinant reporter fusion constructs *35S*:PtobZIP18-GFP. These fusion vectors were transformed into Agrobacterium strain GV3101, and the bacterial suspension (OD600 = 0.8) was infiltrated into one-mouth-old tobacco leaves for 72 h. The fluorescence signals of green fluorescent protein (GFP) were observed using a Leica confocal microscope (FV3000; Olympus, Tokyo, Japan).

## Method S11 Histochemical and histological analysis

The stems of 3-month-old greenhouse-grown plants were fixed in 70% (v/v) formaldehyde-acetic acid (FAA) fixative. After rinsing with 0.1 M cacodylate buffer, the samples were dehydrated through a series of ethanol concentrations (v/v: 60%, 70%, 80%, 90%, and 100%) and then further dehydrated in 100% dimethylbenzene. The samples were embedded in methyl methacrylate, and 10 μm thick cross sections were cut using a rotary microtome (HistoCore AUTOCUT; Leica Microsystems). The sections were stained with 1% (w/v) toluidine blue and examined using an Aperio Versa slide scanner (Leica, Aperio VERSA, Wetzlar, Germany).

## Method S12 Luciferase complementation imaging assays (LCI)

The dual-luciferase reporter transient transactivation assay was performed as previously reported (Zhou *et al*., 2015). Briefly, the CDS of *PtoWRKY19* and *PtobZIP18* were cloned and inserted into the effector pGreenII-62SK. The PtoWRKY19 binding site in *PtobZIP18* promoter and the PtobZIP18 binding site in the promoters of *PtoGATL3*, *PtoCESA3* and *PtoDUF1635* were fused with the firefly luciferase (LUC) reporter gene in the pGreenII-0800-LUC reporter vector. The effector and reporter constructs were co-transformed into tobacco leaves. The permeabilized leaves were then sprayed with a 100 mM D-luciferin potassium salt solution (Biorigin, Beijing, China). Fluorescence imaging was performed using the Molecular imaging system (LB983 NightOwl Ⅱ, Germany). Luciferase activity was measured using the Dual-Luciferase Reporter Assay System (Vazyme) and quantified with a GloMax 20/20 Luminometer. The ratio of LUC:REN was calculated using relative REN activity as the control.

## Method S13 Yeast one-hybrid assay(Y1H)

The promoter sequences of *PtobZIP18, PtoGATL3*, *PtoCESA3*, and *PtoDUF1635* were amplified and inserted into the *pAbAi* vector. The recombinant *pAbAi* vectors were digested with BstBI (NEB, Ipswich, MA, USA), and the linearized plasmids were transformed into Y1H Gold yeast cells to serve as baits. The prey plasmids AD-*PtoWRKY19* and AD-*PtobZIP18* were then transformed into the bait strains. Empty AD-Rec and AD vectors were also transformed into the bait strains as negative controls. Single colonies were selected and cultured on SD/−Leu media supplemented with the appropriate amounts of AbA to confirm positive interactions.

## Method S14 Electrophoretic mobility shift assay (EMSA)

The CDSs of *PtoWRKY19* and *PtobZIP18* were amplified and cloned into the *pET-32a* vector to generate His fusion proteins. The His-*PtoWRKY19* and His-*PtobZIP18* recombinant proteins, containing HIS tags, were introduced into Escherichia coli BL21(DE3) (EC1002, Shanghai Weidi Biotechnology, Shanghai, China). The recombinant proteins were purified using an HIS-tag Protein Purification Kit (P2226; Beyotime Biotechnology, Shanghai, China). Oligonucleotide probes were synthesized and biotin-labeled by Sangon Biotech Company. EMSAs were performed using a Light Shift Chemiluminescent EMSA Kit (GS009, Beyotime) following the manufacturer’s protocol.

## Method S15 SPR‑based intermolecular binding assay (SPR)

SPR measurements were conducted using five dilutions of *PtobZIP18^hap1^* and *PtobZIP18^hap2^* oligonucleotides on a Biacore X100 platform (Wakasugi *et al*., 2024). Purified PtoWRKY19-His proteins were immobilized on CM5 chips (GE Healthcare, Madison, WI, USA). The oligonucleotide dilutions for PtobZIP18hap1 and PtobZIP18hap2 were 0.5363, 1.341, 3.352, 8.38, and 20.95 µM. Manual run and single-cycle kinetics modes were used to determine the response unit (RU) values and dissociation constant (Kd) values, respectively. Data analysis was performed using Biacore X100 evaluation software version 1.0+ (Cytiva).

## Method S16 Chip-qPCR

Chromatin Immunoprecipitation (ChIP) was performed using 5 g of fresh stem segments from 3-month-old PtobZIP18-GFP poplar plants. Tissues were vacuum-infiltrated with 1% formaldehyde for cross-linking, washed with cold water, dried, and ground into powder in liquid nitrogen. Chromatin was fragmented to ~500 bp by sonication and precleared with Protein A beads. Immunoprecipitation was conducted overnight using either anti-GFP antibody (Sigma) or mouse IgG control. The precipitated complexes were washed sequentially with: low-salt buffer, high-salt buffer, LiCl buffer, and TE buffer. After elution, cross-links were reversed with 5 M NaCl. RT-qPCR was then performed on the 7500 Fast Real-Time PCR System using SYBR Premix Ex Taq™ Kit (TaKaRa, Dalian, China) according to the manufacturers’ instructions.

## Method S17 Yeast two-hybrid assays (Y2H)

A cDNA library of *Populus* was constructed using the Oebiotech Yeast Two-Hybrid System (Oebiotech, Beijing, China) following the manufacturer's instructions. *PtobZIP18* was selected as the bait for screening the cDNA library due to its transactivation activity in yeast. For the Y2H assays, the full-length *PtobZIP18* and *PtoCIPK9* genes were cloned into the pGBKT7 and pGADT7 vectors, respectively. The recombinant plasmids were co-transformed into the yeast strain Y2H Gold (YC1002; Shanghai Weidi Biotechnology). Yeast cells containing the recombinant plasmids were cultured on SD-Trp/Leu and SD-Trp/Leu/His/Ade media, with or without x-α-gal, for protein interaction analyses.

## Method S18 Split luciferase (split-LUC) complementation assay

To construct luciferase complementation vectors, the pCAMBIA1300-35S-NLuc vector was fused to the C-terminus of PtobZIP18, and the pCAMBIA1300-35S-CLuc vector was fused to the C-terminus of PtoCIPK9. Transient expression was conducted in *Nicotiana benthamiana* leaves via GV3101 *Agrobacterium* infiltration. The plants were incubated at 22°C for 2 days, after which luciferase activity was measured. Luminescence images were captured using Molecular imaging system (LB983 NightOwl Ⅱ, Germany) imaging system.

## Method S19 *In vitro* phosphorylation assay

The *in vitro* phosphorylation assay was conducted as described previously (Li *et al*., 2017). Recombinant GST-PtoCIPK9 was incubated with HIS-PtobZIP18 in a reaction buffer containing 20 mM Tris-HCl (pH 7.5), 10 mM MgCl₂, 25 mM ATP, and 1 mM DTT at 30°C for 2 h. The reactions were terminated by adding 5× sodium dodecyl sulfate (SDS) sample loading buffer and separated on a 10% SDS–polyacrylamide gel electrophoresis (SDS–PAGE) gel. Phosphorylated signals were detected and visualized using autoradiography with a Typhoon 9410 imager.

## Method S20 LC-MS/MS assay

Purified HIS-PtobZIP18 and GST-PtoCIPK9 proteins were incubated in a protein kinase reaction buffer containing 20 mM MgCl₂, 50 mM Tris–HCl (pH 7.5), 1 mM DTT, and 50 mM ATP at 30°C for 30 min. The reaction products were reduced with DTT, alkylated with iodoacetamide, and subsequently digested with trypsin (pH 8.5) at 37°C for 12 h. The resulting peptides were analyzed by LC-MS/MS following the protocol described previously (Liu *et al*., 2017).

**References**

Du Q, Wang B, Wei Z, Zhang D, Li B. (2012) Genetic diversity and population structure of Chinese White poplar (Populus tomentosa) revealed by SSR markers. *Journal of Heredity*, **103**, 853-862.

Du, Q., Tian, J., Yang, X., Pan, W., Xu, B., Li, B., Ingvarsson, P., Zhang, D. (2015) Identification of additive, dominant, and epistatic variation conferred by key genes in the cellulose biosynthesis pathway in *Populus tomentosa*. *DNA Res*, **22**, 53-67.

Fang, Y., Wang, D., Xiao, L., Quan, M., Qi, W., Song, F., Zhou, J. *et al*. (2023) Allelic variation in transcription factor PtoWRKY68 contributes to drought tolerance in *Populus*. *Plant Physiol.* **193**, 736-755.

Kumar, S., Stecher, G., Tamura, K. (2016) MEGA7: Molecular Evolutionary Genetics Analysis Version 7.0 for Bigger Datasets. *Mol. Biol. Evol.* **33**, 1870-1874

Li, H., Ding, Y., Shi, Y., Zhang, X., Zhang, S., Gong, Z., Yang, S. (2017) MPK3- and MPK6-mediated ICE1 phosphorylation negatively regulates ICE1 stability and freezing tolerance in Arabidopsis. *Developmental Cell*, **43**, 630-642.

Li, S., Lin, Y.J., Wang, P., Zhang, B., Li, M., Chen, S., Shi, R. (2019) The AREB1 Transcription Factor Influences Histone Acetylation to Regulate Drought Responses and Tolerance in *Populus trichocarpa*. *Plant Cell*. **31**, 663-686.

Liu, Z., Jia, Y., Ding, Y., Shi, Y., Li, Z., Guo, Y., Gong, Z., & Yang, S. (2017) Plasma membrane CRPK1-mediated phosphorylation of 14-3-3 proteins induces their nuclear import to fine-tune CBF signaling during cold response. *Molecular Cell*. **66**, 117-128.

Sluiter A, Hames B, Ruiz R, Scarlata C, Sluiter J, Templeton D, Crocker DLAP. (2005) Determination of structural carbohydrates and lignin in biomass. *Lab Anal Proced*. **1617** :1–16.

Trapnell, C., Pachter, L., Salzberg, S.L. (2009) TopHat: discovering splice junctions with RNA-Seq. *Bioinformatics*. **25**, 1105-1111.

Wakasugi, D., Kondo, S., Ferdousi, F., Mizuno, S., Yada, A., Tominaga, K., Takahashi, S. (2024) A rare olive compound oleacein functions as a TrkB agonist and mitigates neuroinflammation both in vitro and in vivo. *Cell Communication and Signaling*. **22**(1), 309.

Wang, C., Liu, S., Dong, Y., Zhao, Y., Geng, A., Xia, X., Yin, W. (2016) PdEPF1 regulates water-use efficiency and drought tolerance by modulating stomatal density in poplar. *Plant Biotechnol. J.* **14**, 849-860.

Yeh, T., Yamada, T., Capanema, E., Chang, H., Chiang, V., Kadla, J. (2005) Rapid screening of wood chemical component variations using transmittance near-infrared spectroscopy. *J. Agric. Food Chem.* **53**, 3328-3332.

Zhou, H., Lin-Wang, K., Liao, L., Gu, C., Lu, Z., Allan, A. & Han, Y. (2015) Peach MYB7 activates transcription of the proanthocyanidin pathway gene encoding leucoanthocyanidin reductase, but not anthocyanidin reductase. *Frontiers in Plant Science*. **6**, 908.
